# Genotype × Environment interactions of Nagina22 rice mutants for yield traits under low phosphorus, water limited and normal irrigated conditions

**DOI:** 10.1038/s41598-018-33812-1

**Published:** 2018-10-19

**Authors:** Yugandhar Poli, Divya Balakrishnan, Subrahmanyam Desiraju, Madhusmitha Panigrahy, Sitapati Rao Voleti, Satendra Kumar Mangrauthia, Sarla Neelamraju

**Affiliations:** ICAR-Indian Institute of Rice Research, Rajendranagar, Hyderabad 500030 India

## Abstract

Multi environment testing helps identify stable genotypes especially for adverse abiotic stress situations. In the era of climate change and multiple abiotic stresses, it becomes important to analyze stability of rice lines under both irrigated and stress conditions. Mutants are an important genetic resource which can help in revealing the basis of natural variation. We analyzed 300 EMS induced mutants of *aus* rice cultivar Nagina22 (N22) for their G × E interaction and stability under low phosphorus (P), water limited and irrigated conditions. Environmental effect and interaction were more significant than genotypic contribution on grain yield (GY), productive tillers (TN) and plant height (PH) under these three environmental conditions in dry season, 2010. GY and TN were more affected by low P stress than by water limited condition, but PH was not significantly different under these two stresses. Mutants G17, G209, G29, G91, G63 and G32 were stable for GY in decreasing order of stability across the three environments but G254 and G50 were stable only in low P, G17 and G45 only in water limited and G295 and G289 only in normal irrigated condition. We then selected and evaluated 3 high yielding mutants, 3 low yielding mutants and N22 for their stability and adaptability to these 3 environments in both wet and dry seasons for six years (2010–2015). The most stable lines based on the combined analysis of 12 seasons were G125 (NH210) under normal condition, G17 (NH686), G176 (NH363) and G284 (NH162) in low P condition and G176 (NH363) under water limited condition. G176 was the best considering all 3 conditions. When screened for 15 *Pup1* gene-specific markers, G176 showed alleles similar to N22. While two other low-P tolerant lines G17 and G65 showed N22 similar alleles only at k-1 and k-5 but a different allele or null allele at 13 other loci. These stable mutants are a valuable resource for varietal development and to discover genes for tolerance to multiple abiotic stresses.

## Introduction

Rice farming is the backbone of agriculture in Asia as over 90% of the world’s rice is produced and consumed in this region. There is a disparity between the rate of rice production and the rate of increase in rice consuming population, leading to more emphasis on improving rice productivity. Breeding rice varieties with tolerance to abiotic stresses is an economically viable and sustainable option to improve rice productivity. Hence there is a necessity to tackle the abiotic stresses which together restrict the ability of rice to realize its complete yield potential. However, improving stress tolerance in crops through conventional breeding is limited due to the complex underlying mechanisms and also high environmental influence. Additionally the phenomenon of climate change is likely to widen the area affected by abiotic stresses. Different abiotic stresses often occur together or one after another in the same crop season. Some of the common abiotic stresses that limit rice crop growth and productivity are drought, salinity, nutrient deficiency and high temperature. Of these, drought and phosphorus deficiency have become major concerns which affect rice production, especially in water limited cultivation systems. Increasing genetic diversity through mutants can generate desirable variability required to cope with abiotic stresses in the coming decades. It is also crucial for the sustainable management of water and fertilizer for agricultural production.

Water is one of the most important components for sustainable rice production and its deficit is a concern in both irrigated and rainfed areas where rice is grown. Rice uses two to five times of the water required by other staple crops such as wheat or maize and uses about 30% of the freshwater consumed for agricultural crops worldwide. Water stress is defined as the lack of sufficient moisture for the normal growth and development^1^. Water is imperative for plant to complete its life cycle and it affects the physiological and biochemical pathways/metabolism such as photosynthesis, ion uptake, carbohydrate translocation and metabolism of nutrients^2^ and prolonged stress can even affect plant survival. To reduce water use in rice cultivation while maintaining its high yield levels, water-saving regimes have been developed. Multi-environment evaluation of agronomic traits under stress condition is a prerequisite to identify highly tolerant rice genotypes for sustainable crop production.

Phosphorus is the second most important macro nutrient that is essential for the entire growth period of any crop. P is required for major processes such as photosynthesis and respiration. It also plays important role in lipid, protein, and nucleic acid metabolism. Majority of the Indian soils have low P availability and to overcome the situation, P fertilizers are supplied externally. Even then the entire applied P does not become available to plants as P is lost in the processes of mineralization, fixation and leaching. In addition, rice crop has very low P use efficiency (~25%)^3^. It has also been predicted that P source rock phosphate is non-renewable and finite, likely to be depleted in the next 50 to 60 years. Farming communities in developing countries face lots of problems to obtain P fertilizers due to its high cost and limited availability. Hence a practical solution is the development of varieties which perform well under low P conditions.

There is a need of strategies which integrate genetic improvement targeting new varieties with improved capacities to efficiently use available nutrients and water resources along with management practices for sustainable global food security^4^. Keeping in view, the prevailing situation of water scarcity and P deficiency, it is essential to identify rice genotypes that are tolerant and give stable yield in response to these abiotic stresses. Identification of suitable genotypes for multiple environments is a challenge in breeding programs. Selection of stable genotypes along with high yield is also essential. Thus effects of G × E interactions need to be considered for evaluation of genotypes for variety development^5,6^. Stability analysis can be conducted on replicated trials over several environments following environment wise analysis of variance and pooled analysis of variance. Several stability statistics have been proposed to estimate G × E. The traditional measures use the coefficient of variation^7^ environmental variance^8^ stability variance^9^ regression based parameters^10^ and stability analysis^11^. AMMI (Additive Main Effect and Multiplicative Interaction) and GGE (Genotype and Genotype × Environment Interaction) biplot are excellent tools for visual data analysis for different environments^12^. In AMMI, the additive portion is analyzed through Analysis of Variance (ANOVA) and interaction effects through the Principal Component Analysis (PCA) model. Biplots helps in displaying genotypic stability statistics and clustering of genotypes based on their performance in different environments^13^. GGE biplot gives more detailed graphical representation of mean values and stability and displays the which-won-where pattern of genotypes. It also identifies mega environments for selection and discriminating test environments^14–19^. The quality of selections was enhanced when stability variance was assessed along with yield or traits in combination with yield^20,21^. This model was effectively used in several crops for G × E studies^22–24^ especially for assessing grain yield.

*Pup1 (Phosphate uptake 1*) is a major rice QTL identified in chromosome 12 for P deficiency tolerance in a population derived from Nipponbare/Kasalath and this QTL enhances the plants efficiency for higher uptake of P from the soil^25^. *Pup1* improves root growth and increases P uptake per root size^26^. Comparative sequence analyses of *Pup1* (278-kbp) region showed significantly different large insertions or deletions (INDELs) linked to low-P tolerance and present in *aus*-type tolerant donor variety Kasalath when compared to *japonica-*type Nipponbare^27^. Markers were developed based on *Pup1* genomic sequence of the tolerant donor variety Kasalath^27^. These were validated in 159 diverse rice accessions and *Pup1* was present in *aus*-type varieties except in N22 which showed only two markers similar to Kasalath^28^. *OsPup*K46-2 was detected as candidate gene closely linked to low-P tolerance and named as phosphorus-starvation tolerance 1 (*PSTOL1*)^28–30^. In our study 65 markers comprising 15 *Pup1* specific markers and 50 SSRs were used for genotyping to find if they were associated with low-P tolerance in mutants.

Approximately 22000 mutants have been generated using chemical mutagen ethyl methane sulphonate (EMS) in the upland rice variety Nagina22 in India under the project ‘Generation, characterization and use of EMS induced mutants of upland variety Nagina-22 for functional genomics in rice’^31,32^. Sixty seven mutants and the parent Nagina 22 (N22) at Indian Institute of Rice Research (IIRR) were evaluated under normal, low P field and alternate wet and dry (AWD) conditions for one season and 6 gain of function mutants with significantly higher grain yield were identified^33^. Three hundred N22 mutants including these 67 were used to identify stable mutants for yield, tiller number and plant height in three environments; normal irrigated, low P and water limited conditions at IIRR farm from 2010 to 2015. Phenotypic data of selected 3 tolerant and 3 sensitive mutants based on yield in low P along with N22 for 12 seasons was then used to analyse G × E interactions.

## Materials and Methods

### Location

The research work was conducted in ICAR-IIRR, Hyderabad during 12 crop seasons (two seasons, *kharif* = wet season and *rabi* = dry season, per year) from 2010 to 2015. The IIRR farm is geographically situated at 17° 19′ N latitude and 78° 29′ E longitude and at an altitude of 542.7 m above mean sea level. The soil is alkaline vertisol with a pH of 7. 94. Weather data during the crop season is given in Table 1. The soil properties are mentioned in Table 2.

**Table 1:** Weather parameters during wet season 2010.

Wet season 2010	Temperature (°C)			Relative Humidity (%)		Rainfall (mm)	Rainy Days	Sunshine (Hrs.)	Wind Speed (Km/Hr)	Evaporation (mm)	Crop stage
	Max.	Min.	Mean Temp.	I	II						
June	35.20	24.70	29.95	82.00	60.00	113.70	6.00	5.70	9.80	5.60	Sowing
July	29.40	22.50	25.95	89.90	77.00	278.90	16.00	2.50	9.20	3.40	Transplanting
Aug	30.30	22.60	26.45	91.00	75.00	203.10	13.00	4.40	5.60	2.90	Vegetative stage
Sep	29.40	22.20	25.80	89.00	71.00	243.80	13.00	4.10	4.90	2.70	Anthesis
Oct	30.10	20.60	25.35	86.00	63.00	108.00	7.00	5.80	3.00	2.50	Harvesting
Mean	30.88	22.52	26.70	87.58	69.20	189.50	11.00	4.50	6.50	3.42

**Table 2:** Soil properties of three plots Low P (E1), Water limited (E2) and Normal (E3).

Season	Soil property	Low P (E1)	Water limited (E2)	Normal (E3)
Wet season 2010	Soil type	Clayey Vertisol	Clayey Vertisol	Clayey Vertisol
	pH	7.60	7.80	7.80
	Electrical conductivity (dS/m)	0.22	0.24	0.24
	Organic carbon (%)	0.52	0.54	0.54
	Available N (kg/ha)	210.00	210.00	210.00
	Available P_2_O_5_ (kg/ha)	1.80	24.00	24.00
	Available K_2_O (kg/ha)	310.00	310.00	310.00

### Plant material

**Figure 1:**
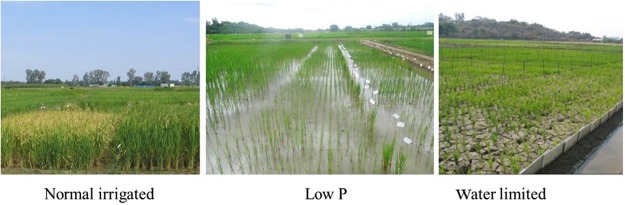
Rice experimental plots of normal irrigated, low P, and water limited field conditions at IIRR, Hyderabad, India.

A set of 300 (M4 generation) mutants advanced from ethyl methane sulfonate (EMS) induced N22 mutants^34^ were used in this study along with its wild type (N22) as control (Supplementary Table 1). These mutants were grown in nursery beds for 25 days and then transferred into plots of three environments in randomized complete block design (RCBD) with 3 replications. Based on grain yield in low P field, 3 gain of function mutants (gof) (G17-NH686, G65-NH787, G176-NH363) with higher grain yield than N22 and 3 loss of function (lof) mutants (G284-NH162, G125-NH210, G173-NH359) with lower grain yield than N22 were selected. These six mutants were screened along with N22 in, low P, water limited and normal irrigated (hereafter referred as normal) conditions (Fig. 1) for 12 seasons including six *kharif* (wet) and six *rabi* (dry) seasons with five replications in RCBD.

### Experiment details

#### Environment 1: Low P condition

Low P plot has been maintained without external application of P fertilizers from 39 years^35^. The available P is very low (Olsen P 1.8 kg/ha) in this soil environment. Water level was maintained at a depth of 3 cm at initial stages and increased to 5 cm at panicle initiation stage upto physiological maturity.

#### Environment 2: Water limited conditions

The field was saturated with water for 10–15 days at the time of transplanting, after which irrigation was not given for 15 days. The field was later re-irrigated when cracks appeared in the field till the required level of water was attained. Again irrigation was stopped till the appearance of cracks in the soil. This cycle was repeated till physiological maturity. The mutants were subjected only to a moderate level of water stress. Double bunds and asbestos sheets were kept surrounding the water limited plot to restrict water inflow and outflow.

#### Environment 3: Normal irrigated condition

25 days old seedlings were transplanted in RCBD with one seedling per hill and 21 plants in each row and the spacing was 15 × 20 cm. The field was maintained continuously in flooded conditions. The water level was maintained at a depth of 3 cm at initial stage (transplanting) and increased to 5 cm at panicle initiation stage up to physiological maturity. There was no mid-season drainage.

In all the three plots nitrogen (urea) and potassium (muriate of potash) were applied at 100:60 kg/ha. In water limited and normal plot, P was applied as SSP (single super phosphate) at the rate of 60 kg/ha as basal dose but not in low P plot. The entire K, 50% N and 12.5 kg/ha zinc (zinc sulphate) was applied as a basal dose in all the three plots. The remaining 50% N was applied as two splits, 25% N at tillering stage and 25% at booting stage. In each row 21 plants with one seedling per hill were transplanted with a spacing of 15 × 20 cm.

### Morphological observations

Genotypes were screened for grain yield, plant height and tiller number in three environments following Standard Evaluation System (n = 5)^36^. Plant height was recorded at the time of flowering by measuring length from the base of the plant to the tip of the main panicle and excluding awn if present and was expressed in cm. Tiller number per plant was recorded after anthesis. Grain yield (g) per plant was recorded from harvested panicles at physiological maturity. Grains were threshed, cleaned and sun dried until the moisture content was consistent at 14%.

### Statistical analysis

Analysis of variance (ANOVA) was calculated for individual environments and then a combined analysis of variance was estimated, and environments and genotypes were considered as fixed using PB tools (Version 1.4, http://bbi.irri.org/products) and R^37^. Analysis of variance was done individually with each trait using AMMI biplot and GGE biplot models with PB Tools ver.5. These results were used to study G × E interaction. The graphs generated were based on (1) AMMI biplot for yield, (2) GGE biplot environment view for yield (Evaluation of genotypes relative to ideal genotypes), (3) Polygon view of GGE biplot for identification of which won where. The stability variance was incorporated with yield to obtain the YSi statistic for the selection of high yielding and stable genotypes simultaneously^38^.

#### Two way analysis of variance

Two way analysis of variance was carried out for each character in order to determine genotype × environment interaction. Further the data with significant interaction was subjected to stability analysis.

#### Stability analysis

The results of AMMI model^12^ for analyzing the stability and interaction are mainly interpreted using AMMI1 biplot plotted with the genotypes and environments main effect and first multiplicative axis term (PC1). The values of Principal Component Axis (PC1) scores, either negative or positive, are proportional to the specific adaptation of a genotype to the environments. The stability of the genotype over the environments is determined on approximation of PC1 scores to zero. In the text the genotypes are mentioned in the order of their decreasing stability.The AMMI is a combined model of analysis of variance (ANOVA) and principal component analysis (PC). The AMMI model equation^14^ was written as:

$${Y}_{ij.}=\mu +{\delta }_{i}+{\beta }_{j}+\sum _{k=1}^{K}{\lambda }_{k}{\delta }_{ik}{\beta }_{jk}+{\varepsilon }_{ij.}$$

Both G and GE variation were graphically represented by GGE biplots^15^. It uses sites regression (SREG) linear–bilinear model as given in the formula

$${Y}_{ij.}=\mu +{\beta }_{j}+\sum _{k=1}^{K}{\lambda }_{k}{\delta }_{ik}{\beta }_{jk}+{\varepsilon }_{ij.}$$

where Y(ij.) is the mean of i^th^ genotype in j^th^ environment, µ is the overall mean, δi is the genotypic effect, βj is the environment effect, λk is the singular value for PC axis k: δik is the genotype eigenvector value for PC axis n, βjk is the environment eigenvector value for PC axis k and εij is the residual error assumed to be normally and independently distributed. GGE biplots were used to estimate (1) mega environment analysis (which-won-where pattern), for recommending specific genotypes to specific mega environments. (2) Genotype evaluation for determining stable genotype(s) across the locations and (3) location evaluation, for understanding discriminative power among genotypes in target locations.

### Genotyping

Genomic DNA was isolated from fresh leaf samples of N22 and 6 selected mutants; G17, G65, G125, G173, G176, G284, adopting CTAB method^39^. In all, 65 markers (15 *Pup1* related and 50 SSRs) were used for genotyping (Supplementary Table 2)^26–30,40^. PCR was conducted using 10 µl reaction mixture containing 15 ng of genomic DNA, 1X assay buffer, 200 μM of dNTPs, 1.5 mM MgCl_2_, 10pmol of forward and reverse primer and 1 unit of Taq DNA polymerase (Thermo Scientific) with thermal cycler (G-STORM, USA). PCR steps were programmed as initial denaturation for 5 min with 94 °C followed by 35 cycles at 94 °C for 30 sec, 55 °C for 30 sec, 72 °C for 1 min with a final extension for 10 min at 72 °C. Amplified PCR products were resolved in 3% agarose gel prepared using 0.5 X TBE buffer and electrophoresis was carried out at 120 V for 2 h. Ethidium bromide stained gels were documented with Alpha Imager, (USA) gel documentation system.

### Single marker association analysis

Genotypic data using 41 polymorphic SSR markers on all chromosomes were subjected to MAP function using the Kosambi mapping function^41^. Single marker analysis was carried out using SMA method in IciMapping v4.1 (www. isbreeding.net).

## Results

### Yield and yield related traits in E1 (low P), E2 (water limited) and E3 (normal condition)

Grain yield, tiller number and plant height were significantly higher in E3 compared to E2 and E1 (Fig. S1). Grain yield ranged from 0 to 8.38g in E1, 0.69 to 29.44g in E2 and 3.55 to 27.79g in E3. Tiller number ranged from 1 to 20 in E1, 3 to 25 in E2 and 6 to 26 in E3. The plant height ranged from 41 to 88 cm in E1, 39 to 90 cm in E2 and 72 to 127 cm in E3 (Fig. S2). Across all the environments G17 showed highest grain yield, G275 had highest tiller number and G217 had highest plant height. 150 mutants showed consistent performance for higher yield than N22, 148 genotypes for tiller number and 149 genotypes for plant height in all 3 environments. N22 was ranked for performance across environments, for yield it was 151^st^, for tiller number 149^th^ and for plant height 150^th^ position. Among the top 50 genotypes in all the three environments for each trait, there were 9 genotypes G301 (NH123), G277 (NH277), G276 (NH218), G274 (NH152), G218 (NH109), G139 (NH8), G111 (NH430), G20 (NH552) and G19 (NH649) which performed well for all the three traits; high yield, tiller number and plant height. 27 of the top 50 genotypes showed both high yield and high tiller number and 18 genotypes had both high tiller number and plant height.

In E1, 109 mutants had higher and 81 had lower average grain yield compared to N22. In E2, 112 mutants had higher grain yield than N22 and 162 had lower yield. Similarly in E3, 124 mutants and 145 mutants showed higher and lower mean yield levels respectively compared to N22. In case of tiller number, compared to N22 in E1; 166 mutants had a higher tiller number and 79 had lower tiller number. In E2; 154 mutants had higher tiller number than N22 and 121 had lower. In E3; 94 and 120 mutants had higher and lower tiller number than N22, others showed similar phenotype as N22. In E1, 200 mutants had higher plant height than N22 and 81 mutants had lower. In E2 30 mutants had higher plant height and 265 mutants had lower. In E3 142 mutants had higher plant height and 142 had lower plant height than N22.

### Stability of genotypes across the environments

#### Grain yield

**Figure 2:**
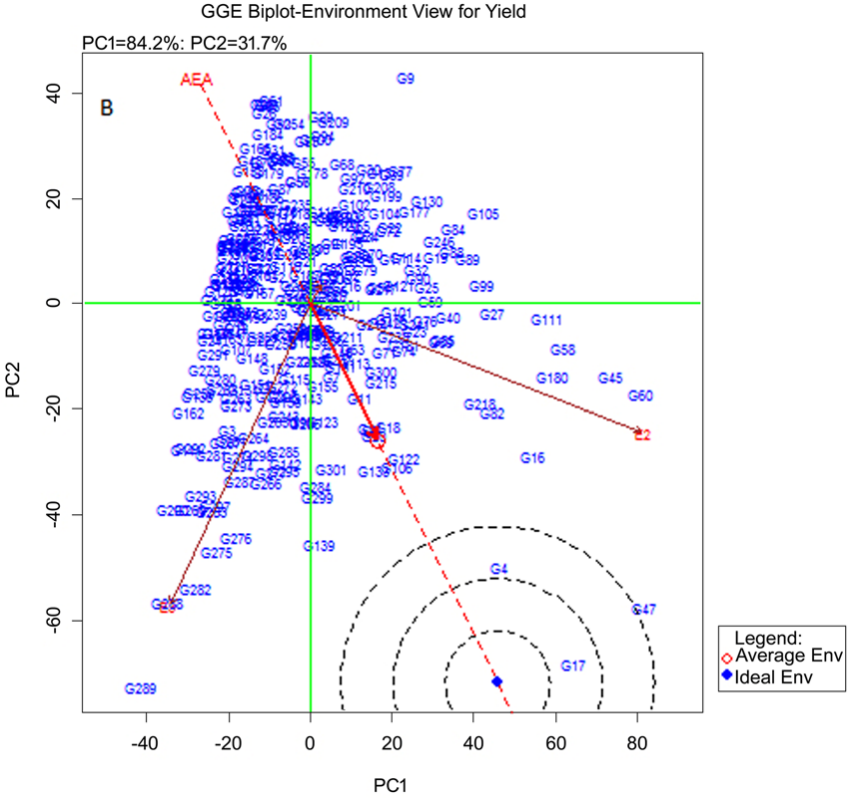
GGE biplot of 300 mutants and N22 compared to ideal genotype for yield traits in 3 environments E1 (low P), E2 (water limited) and E3 (normal irrigated).

300 mutants in 3 environments for 1 season: Combined ANOVA for the data on mutants in three environments indicated significant variance and mean sum of squares for genotype as well as for genotype × environment effect (Table 3A). Across all the environments, genotypic (G) effect was 11% for grain yield, environment (E) effect was 54%, and genotype and enivironment (G × E) effect was 34%. For tiller number, G effect was 23%, E effect was also 23% and G × E was 53% (Table 3B). For plant height G effect was 8%, E effect was 73%, G × E effect was 18% (Table 3C).The interaction between G × E was highly significant in case of grain yield. Among the environments E3 was the most suitable environment for expression of yield potential. The genotypes G209, G29, G91, G63 and G32 were highly stable across all the three environments followed by G51, G55, G91, G206, G132, G191, G95 and G186 in decreasing order of stability (Fig. 2). These genotypes showed more of additive effect for the traits study than the remaining genotypes. The genotypes G107, G22 and G9 showed more interaction effect than genotypic effect. There was a strong interaction between the environments and genotypes. G289 and G17 were the most responsive genotypes. Genotypes G254, G50, G191, G29, G104 and G91 were most suitable for E1, G17 and G45 for E2 and G289 for E3 considering yield. The most adaptable genotype in each environment was G254 in E1, G17 in E2 and G289 in E3 which were also high yielders in the respective environments (Fig. S3).

**Table 3:** Analysis of variance of plant height, tiller nnumber and grain yield in 300 mutants and N22 in three environments.

	df	Sum of Squares	Mean Squares	F	p value	SS (%)
**A. Analysis of variance of plant height in 300 mutants and N22 in three environments**						
Total	902	658866.00				
Genotypes	300	55920.52	186.40	0.92	0.78	8.48
Environments	2	481915.20	240957.60	8251.97	<0.001	73.14
Interaction	600	121030.30	201.71	6.91	<0.001	18.36
Heterogeneity	300	58556.29	195.18	0.94	0.71	8.88
Residual	300	62474.00	208.24	7.13	<0.001	9.48
Pooled error	1800		29.20			
**B. Analysis of variance of tiller number in 300 mutants and N22 in three environments**						
Total	902	38041.49				
Genotypes	300	8851.10	29.50	0.87	0.91	23.26
Environments	2	8863.52	4431.76	1877.86	<0.001	23.29
Interaction	600	20326.86	33.87	14.36	<0.001	53.43
Heterogeneity	300	7471.29	24.90	0.58	1	19.63
Residual	300	12855.57	42.85	18.16	<0.001	33.79
Pooled error	1800		2.36			
**C. Analysis of variance of grain yield in 300 mutants and N22 in three environments**						
Total	902	80008.44				
Genotypes	300	8896.71	29.65	0.65	1	11.11
Environments	2	43640.59	820.29	21184.75	<0.001	54.54
Interaction	600	27471.14	45.78	44.45	<0.001	34.33
Heterogeneity	300	8928.34	29.76	0.48	1	11.15
Residual	300	18542.80	61.80	60.01	<0.001	23.17
Pooled error	1800		1.03

Thus, G17 is potential donor in crop improvement programs for yield and tiller number improvement at low P, water limited and normal conditions. G17 gives 7g mean single plant yield in low P and 24g in water limited condition than compared to 0.89g and 8.35g respectively of N22. G17 was also tested under multiple abiotic stress Plant Physiology trials in different locations throughout India in AICRIP (All India Coordinated Rice Improvement Program). It ranked 3^rd^ among 10 genotypes tested for tolerance to multiple abiotic stresses (salt, water, cold and anaerobic conditions). This multilocation data lends support to our finding that G17 is the best mutant among 300 tested and can be used as a stress tolerant variety or a stable donor for tolerance to water or low-P stress.

**Figure 3:**
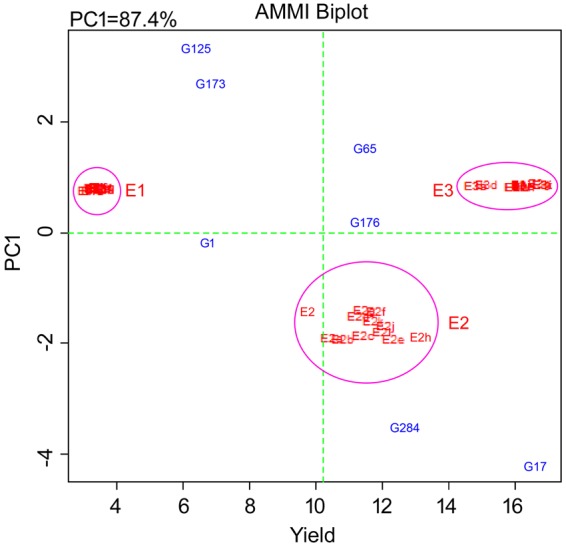
AMMI1 biplots for yield of 6 mutants and N22 tested in three environments over 12 seasons.

Evaluation of 6 mutants and N22 in 3 environments for 12 seasons: Significant differences were observed between treatments and mutants for grain yield by combined environment evaluation across 12 seasons. The mean grain yield was significantly more in normal condition compared to low P and limited water condition. Grain yield was higher in the dry seasons compared to wet seasons in low P condition, but was reverse in normal condition. In water limited condition, differences between wet season and dry season were not significant. Compared to all the years, grain yield was higher in low P, water limited and normal conditions in both wet and dry seasons of 2014. The differences were significant between year × environment × genotype × season. In low P condition, G1 (N22), G17 (NH686), G176 (NH363) and G284 (NH162) were the most stable genotypes with G17 and G176 yielding highest (Fig. 3). In water limited condition, only G176 was stable across the environments and all seasons. G17 and G176 showed highest grain yield in E2. In E3, N22 and G176 were most stable and G17, G176 and G173 gave highest yield. Compared to E3, the percent reduction was highest by 94% in G173 and least by 58% in G17 in E1 condition. In E2, maximum reduction of 86% was observed in G65 however G17 showed 43% increase in grain yield compared to E3.

#### Tiller number

Evaluation of 300 mutants in 3 environments for 1 season: In case of tiller number, interaction with environments was highly variable for each genotype. The genotypes G235, G58, G78, G91, G9, G44 and G191 had significant differences among them for tiller number. Genotypes G132, G162, G222, G107 and G51 were best suitable for E1; G17, G16, G45, G111 and G206 for E2 and G275, G297, G287, G295 and G139 for E3. Among the 3 environments, E1 showed severe stress effect among the genotypes and strong G × E interactions were observed. G45 and G17 were the most responsive genotypes. G206, G55, G63, G60, G191 and G195 were stable genotypes for tiller number across the environments. G254 was the best genotype for tiller number in E1, G17 in E2 and G132 in E3 (Fig. S4). Thus, G9 and G17 was a high yielder and with high tiller number in E1 (low P) and in E2 (water limited condition) respectively. G107 and G132 were also high yielding with high tiller number in E3.

Evaluation of 6 mutants and N22 in 3 environments for 12 seasons: Significant differences were observed between environments and mutants for tiller number. Compared to E1 and E2, the mean tiller number was significantly greater in E3. G17 had the highest tiller number in E1, G17 and G176 in E2 and E3. Compared to normal condition, highest reduction of TN was by 81% in N22 and G173 and least reduction by 52% in G17 at low P condition (E1). On the other hand highest reduction was by 46% in N22 and least by 10% in G17 in E2 (water limited condition).

Tiller number was higher in wet seasons than in dry seasons in all the mutants and N22 in low P and normal condition. However, TN was not significantly different between wet and dry seasons in water limited condition. The differences were significant between year × condition, year × genotype × season, year × condition × season.

#### Plant height

Evaluation of 300 mutants in 3 environments for 1 season: E3 was suitable environment for expression of plant height. G121 and G111 were stable in all environments and showed only main effect (additive effect). G217 and G153 were most suitable for E3. Strong interactions were observed between the treatments. Here G44 and G177 were the most responsive genotypes showing highest G × E interaction effects and showed specific adaptation to environments. G121, G111, G222, G29, G206 and G195 were the stable genotypes across the environments. G24 and G49 were best in E1, G255 and G110 in E2 and G217 in E3 for plant height (Fig. S5).

Evaluation of 6 mutants and N22 in 3 environments for 12 seasons: The mean plant height was significantly more in normal condition compared to low P and limited water condition. In all environments, compared to dry season plant height was significantly higher in wet season for all the mutants and N22. Highest plant height was observed in low P, water limited and normal conditions both wet and dry seasons of 2011 and 2012 compared to all the years. The differences were significant between year × environment, year × genotype, year × environment × genotype, year × environment × season. G176 showed highest value for plant height in all conditions. In E1 the largest percent reduction in plant height was in G173 by 53% and least in G17 by 10%, compared to height in E3. On the other hand in E2 largest reduction was in G173 by 40% and least in G17 by 6% compared to height in E3. Thus, G17 showed the least and G173 the most adverse effect on plant height in both low P and water limited condition.

#### Shoot dry weight

Evaluation of 6 mutants and N22 in 3 environments for 12 seasons: Significant differences were observed between treatments and mutants. The mean shoot dry weight was significantly more in E3 compared to E1 and E2 and in wet season compared to dry seasons. Compared to all the years, for E1 condition, both wet and dry seasons of 2011 and 2015 showed high shoot dry weight. For E2 condition, high shoot dry weight was observed in 2011 and 2012 and in 2010 for E3. The differences were significant between year × environment, year × genotype, year × environment × genotype, year × environment × season. G17 and G65 had the highest shoot dry weight in E1 and E2 and G17 and G176 in E3. Compared to E3, highest percent reduction in shoot dry weight was in G173 by 74% and least was in G17 by 62% in E1. Similarly, the largest reduction was in N22 by 42% and least was in G17 and G65 by 32% in E2.

Adaptability of mutants across the environments: For selecting ideal genotypes for each trait, environment view of GGE biplot showed that G17 is ideal for yield and tiller number in all the environments. G4 was next to G17 and may be considered as a desirable genotype for yield. G153 and G8 appeared to be ideal across the environments for height. The polygon view of GGE biplot or which- won- where graph showed that G9 was best suited to E1. G17 followed by G47 were best suited to E2. These two genotypes also showed high yield in E2. G289 was best suited to E3. For tiller number G54 was best suited for E1, G45 and G17 were stable and best suited to E2 and G289 was best suited for E3. G209 and G116 had low tiller number in all the environments. G30 and G194 were the best genotypes for plant height in E1, G285 in E2 and G153 in E3. G286 had low plant height in all the environments. For yield both PC1 and PC2 together account for 100% phenotypic variation. Higher PC1 score is considered as a stable trait across the environments. PC1 for yield was higher than PC2. The first two principal components explained 100% of variation for tiller number but compared to yield PC1 score for tiller number was higher and PC2 score was lower; for plant height PC1 was further lower and PC2 was higher which indicates increase in G × E interaction.

**Figure 4:**
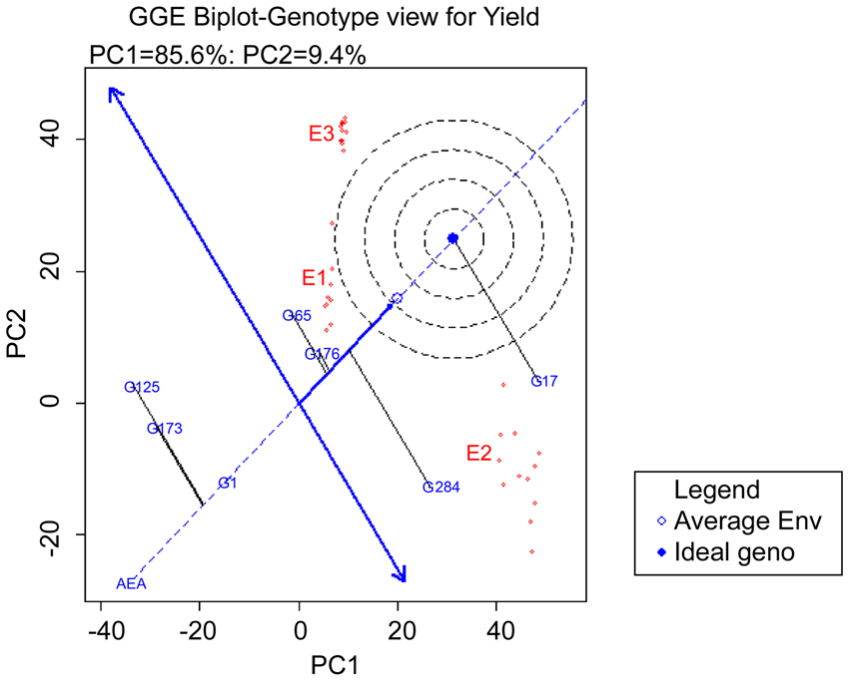
GGE biplot of the 6 genotypes and N22 in comparison with ideal genotype.

**Figure 5:**
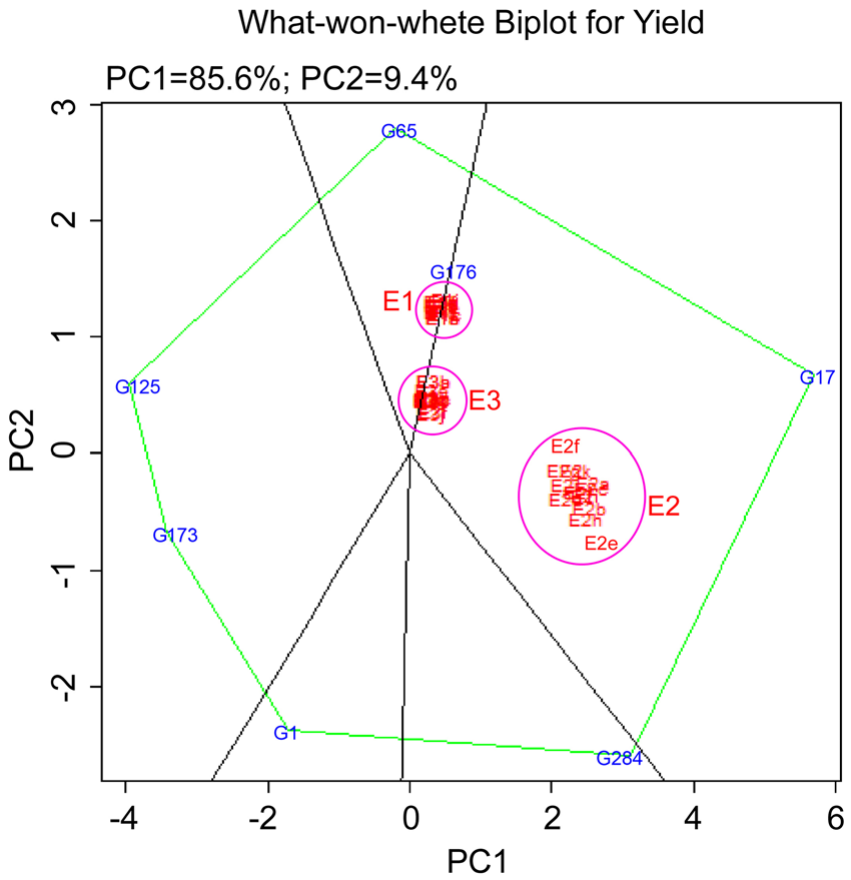
GGE biplot of 6 genotypes and N22 for grain yield in 36 environments based on which-won- where pattern.

GGE biplot genotype view for yield to evaluate stable genotypes showed that G17 is ideal genotype for yield in all the seasons followed by G284, G176 and G65 (Fig. 4). The polygon view of GGE biplot (which won where graph) also showed that G17 is best adapted genotype for all three environments across seasons. Which won where graph, showed that G65 and G176 are suited for both E1 and E2 (Fig. 5).

YSi statistic: YSi statistic analysis is useful for the selection of high yielding genotypes for specific environment and also identification of stable genotypes across different environments which can be used as donors in breeding programs or in multi location trials for varietal release. Based on the YSi value of 300 mutants and N22 during wet season 2010, the top ranked stable 10 mutants were G17, G47, G4, G60, G16, G45, G139, G58, G180 and G82 for grain yield, G287, G295, G275, G297, G17, G111, G20, G16, G139 and G45 for tiller number and G107, G217, G153, G222, G38, G279, G7, G111, G253 and G94 for plant height.

YSi statistic showed that, G17 was the most stable genotype for GY followed by G47, G4, G60, G16, G45, G139, G58, G180 and G82 in the selection ranks based on combined yield and stability analysis. Another 140 genotypes showed high average yield with desirable non- significant stability variance and so they can be considered for evaluation in multi location trials for high yield. N22 was in 158^th^ position in yield and its rank was 144 for yield stability. Similarly for tiller number G275 was the most stable mutant, followed by G287, G297, G17, G111, G295, G16, G139, G20 and G45 based on combined yield and stability analysis. Another 138 genotypes showed high average tiller number with a non-significant stability variance and may be used as donors for enhancing only tiller number. N22 was in 196^th^ position for tiller number and its rank was 98 for stability. Similarly for plant height G217 is the most stable mutant followed by G153, G38, G7, G107, G253, G94, G218, G222 and G245. Another 138 genotypes with non-significant stability variance showed high average plant height while N22 was in 122^nd^ position with a rank of 168 based on combined analysis. G275 with highest tiller number, ranked 37^th^ for yield and 170^th^ for plant height in stability ranking across the environments. G217 ranked 87^th^ for yield 68^th^ for tiller number while N22 ranked 143^th^ for grain yield, 97^th^ for tiller number and 175^th^ for plant height.

In wet season 2010 genotypic (G) effect was 11% for grain yield, 54% for environment (E) effect, and 34% for genotype and enivironment (G × E) effect. For tiller number, G, E and G × E effects were 23%, 23% and 53% respectively. For plant height, G, E and G × E effects were 8%, 73% and 18% (respectively Table 3C). Considering the yield data of 300 mutants across three environments in wet season 2010, genotypic (G) effect percentage was lower and environmental effect and interaction were more significant than genotypic contribution on grain yield (GY), productive tillers (TN) and plant height (PH).

Selected mutants across 12 seasons for combined analysis of 3 environments also showed high environment effect with significant G, G × E and E effect on grain yield. However grain yield data on individual environment across the 12 seasons showed that genotypic effect is greater than environment and interaction effect. Both stress conditions showed higher contribution of G effect (>97%) followed by G × E and E effect but in normal irrigated condition G effect was reduced to 64% followed by E and G × E interaction effects. The deviation in the effects between environments and within individual environment was mainly due to extreme variations in expression of traits in stress and non stress conditions. Variation in genotypic effect in stability analysis between the sets of 300 mutants and selected 6 mutants was mainly due to the difference in number of genotypes, replications and number of environments. The selection of extreme phenotypes which are in the 6 mutants study might have also played a role in difference in genotype, environment and genotype × environment interaction effects as the other set included a large set of genotypes with a performance ranging between the selected lines.

In low P condition, the genotypic (G) effect for grain yield was 99%, environment (E) effect was 0.2% and genotype × environment (G × E) interaction was only 0.2% (Supplementary Table 3A). In water limited condition, G, E and G × E effects were 97%, 1.1% and 1.6% respectively (Supplementary Table 3B). In normal condition, G, E and G × E effects were 65%, 30% and 4.6% respectively (Supplementary Table 3C). In the combined analysis of low P, water limited and normal conditions, G effect was 24%, E effect was 52% and G × E interaction effect was 23% (Supplementary Table 3D). Four mutants G17, G16, G139 and G65 appeared among the top-ranking mutants for both grain yield and tiller number (Supplementary Table 4). Based on *YSi* statistics of 12 season data of selected 6 mutants and N22 in low P condition, G17 had highest rank, followed by G176 and G65 (Supplementary Table 5). Likewise, in water limited condition G4 had highest rank followed by G2, (Supplementary Table 6), and in normal condition G17 and G65 had high ranks (Supplementary Table 7). In combined stability analysis of all three environments; low P (E1), water limited (E2) and normal conditions (E3); G176 was placed in topmost rank followed by G17 and G65 (Supplementary Table 8). The present study also helped in identifying the trait expression particularly suitable for specific environments. Tiller number and yield were stable traits across all the environments in the present experiment. In stress environments E1 and E2, mutants that gave higher yield also had higher tiller number. This implies that along with yield, tiller number also may be considered for identification of stable high yielding genotypes under stress.

Genotyping and single marker analysis: Mutants and N22 were genotyped with 15 *Pup1* specific markers. Two gain of function (gof) mutants G17 and G65 showed allelic similarity between themselves in K1 and K5 loci while remaining 13 markers did not amplify. G176 showed allelic similarity with N22 in all 15 markers, whereas G173 (NH359) and G125 (NH210) showed allelic similarity with N22 in only 6 markers. Among 50 random SSRs used for genotyping, 41 polymorphic markers were identified and tested for allelic variation. Single marker analysis using genotypic data of 41 random markers showed that RM423 was significantly associated with tiller number in water limited and normal conditions (Fig. S6). gof mutants G17 (NH686) and G65 (NH787) and lof mutants G284 (NH162) and G125 (NH210) showed different allelic pattern for RM423 compared to N22, but lof G173 (NH359) and gof G176 (NH363) showed similar alleles as in N22. Two markers RM72 and RM584 were significantly associated with grain yield in low P condition. Lof mutants G284, G125 and G173 showed similar alleles as in N22 but gof mutants G17, G65 and G176 had a different RM72 allele. Likewise, alleles at locus RM584 were similar in lof mutants and N22, but different in gof mutants.

## Discussion

Identification of suitable genotypes for multiple environments is the challenging aspect in breeding programs. Selection of high yielding stable genotypes is essential in any crop breeding program. Consideration of effects of G × E interactions is very important for efficient breeding and evaluation of genotypes for variety development^5,6^. AMMI and GGE biplot are excellent tools for visual data analysis for different environments^12^. In AMMI, the additive portion is analyzed through analysis of variance (ANOVA) and interaction effects through the principal component analysis (PCA) model. Biplots helps in displaying genotypic stability statistics and clustering of genotypes based on their performance across diverse environments^13^. GGE biplot gives more detailed graphical representation of mean performance and stability and displays the which-won-where pattern of genotypes. It also identifies mega environments for selection and discriminating test environments^14–17^. The quality of selections was enhanced when stability variance was assessed along with yield^20^. This method was successfully used in several crops including rice especially for assessing grain yield^42,43^.

In our study, both AMMI and GGE biplot analysis showed that G17 is ideal for E2 considering grain yield. GGE ideal genotype graph showed G17 is ideal for all the environments. Thus, G17 can be used as donor in plant breeding programs for yield and tiller number improvement at low P, water limited and normal conditions. G17 was also tested in multiple abiotic stress Physiology trials in different locations throughout India in AICRIP 2016 (All India Coordinated Rice Improvement Program 2016), G17 performed well across all the locations and it was ranked 3^rd^ in tolerance to multiple abiotic stress. This data supports G17 as the ideal mutant to be used as donor for stable tolerance to abiotic stress or can be improved as a stress tolerant variety. G9 was a high yielder and with high tiller number in low P so it can be used as check while screening the mutants/ genotypes in low P condition. G107 and G132 were best suited to normal irrigated condition so they may be used as reference N22 mutants in normal conditions. It was interesting to note that G17 and G284 showed higher yield under water limited conditions than under normal irrigated conditions. Enhanced yield levels have been reported in water limited conditions previously^44,45^. The response depends on the level of water stress and also the genotypes used. Limited irrigation with moderate soil drying helps in enhanced root growth, root oxidation activity, and photosynthetic rate. This also improves activity of key enzymes involved in sucrose-to-starch conversion in grains resulting in higher grain yield and water use efficiency^46^. Increase in cytokinin levels in the shoot in moderate drying causes greater grain-filling rate and a heavier grain weight^47^. However severe drying causes decreased cytokinin levels and are genotype dependent. Tiller number and yield were stable traits across all the environments in the present experiment. Mutants that gave higher yield also had higher tiller number in stress environments E1 and E2. This implies that along with yield, tiller number also can be considered for identification of stable high yielding genotypes under stress. Usually, high tiller number serves as a surrogate for improved root traits.

Environment effect was high in case of grain yield and plant height but interaction effect was high for the expression of tiller number. Comparatively higher contribution of environment effect was observed in many previous studies^48–51^ and especially under stress conditions^52–54^.

GY and TN were severely affected by low-P stress than by water limited condition, but PH was not significantly different under these two stresses. Interestingly it was observed that TN was most affected in low P condition but PH reduction was observed more under water limited condition. Plant height decline could be attributed to cell enlargement reduction and leaf senescence under water stress^55^. Reduced plant height in response to water stress has been reported earlier also^56,57^. Water limitation showed comparatively less effect than low P stress on shoot growth reduction. There are several reports on biomass reduction due to phosphorus deficiency especially in case of shoot biomass for the benefit of the root system^58–60^. It demonstrates that the plants adapt to different mechanisms to survive and reproduce under various abiotic stresses. Rice cultivars were evaluated at two different locations in glass house and field conditions to dissect the physio-morphological characteristics associated with crop establishment and early vigour of direct seeded rice under drought and P deficiency conditions and found that quick germination and seedling vigour with quick root anchorage and great nutrient uptake capacity, are the important traits for moisture and nutrients stress conditions^61^. A significant interaction between genotype and water regime was observed for plant height and primary root number. However genotype × P interaction was not significant for all traits except variations observed for the total root length, root dry weight and shoot P uptake.

From AMMI analysis, it was inferred that E1 and E2 were not suitable environments for expression of complete yield potential, compared to irrigated condition E3. Compared to E1 and E3, E2 showed more interaction effect. The genotypes which performed better in E1 and E2 environments may be used as reference genotypes to screen for stress specific adaptations. Grain yield of mutants was more stable than plant height and tiller number across the environments. In all the environments, genotypic effect was high for yield and it gradually decreased for tiller number and plant height. Number of mutants which performed better than N22 in stress conditions was more in case of tiller number than for grain yield. But number of mutants with better performance than N22 was more in low P than water stress conditions for plant height.

Single marker analysis using yield traits in three environments with genotypic data using random markers showed that RM423 was associated significantly with tiller number in water limited and normal conditions. Previously, QTLs were identified at RM423 for 1000 seed weight in mapping study using introgression lines from *Oryza rufipogon*^62^. A candidate gene OsMADS29 for rate of germination was detected 10.4 Kb from RM423^63^. The leaf senescence causing allele from N22 mutant was also found at RM423 locus and is an important trait contributing to heat tolerance by retaining chlorophyll content and thereby photosynthetic efficiency^64^. Another interesting causal gene for a lethal mutant of rice with chloroplast development defect was also mapped close to RM423 with a 146Kb distance and this nuclear gene codes for chloroplast 50S ribosomal protein L21 (RPL21c) and its expression is regulated by light^65^.

In our study two SSRs, RM72 and RM584 showed association with grain yield in low P condition (Supplementary Table 9). These markers were reported as linked to yield related traits in previous QTL mapping studies under normal irrigated conditions^66–70^. It was interesting to observe the presence of novel alleles at *Pup1* loci in low P tolerant mutants in two stable high yielding mutants G17 (NH686) and G65 (NH787) and needs further investigation to find the causal mutations. *Pup1* specific markers did not show allelic similarity to Kasalath in amplification pattern of N22 except two markers and others showed no amplification in N22. Various contradictory results have been presented for presence of *Pup1* locus in N22, a drought tolerant popular upland *aus* variety released in the 1920s from Northern India. Chin *et al*., 2009 reported that N22 has only a few Kasalath alleles (*Pup1*-K41 and *Pup1*-K48) at *Pup1* in contrast to other *aus* varieties. In another study Tyagi *et al*., 2012 carried out genotyping using 6 *Pup1* linked markers and found that N22 accession used in their study carried non-Kasalath alleles. Pandit *et al*., 2017 investigated the presence of four gene specific markers *Pup1*-K41, *Pup1*-K42, *Pup1*-K46 and *Pup1*-K59 along with two closest flanking markers RM28073 and RM28102 and found N22 to be positive for all loci. More than 8 accessions and variants of N22 are available in IRRI and NRRI Gene banks (Pandit *et al*., 2017). The disagreement in results of N22 *Pup1* allele may be due to the use of different accessions of N22 in various studies. More detailed analyses of a larger number of replicates and especially comparative analysis of the eight different N22 accessions and the variants are required to confirm the exact status of *Pup1* in N22.

Mutants are very appropriate materials to identify novel variants which can perform in extreme conditions. Mutants are to be studied for their stable performance for yield and the agronomic traits before utilizing in crop improvement programs or the varietal trials. In this study, some of the mutants showed higher tiller number but with low yield and vice versa. Previously, 300 EMS induced N22 mutants were screened in low P field and from them 8 selected mutants were studied further in hydroponics^34^. They concluded that hydroponics could be a surrogate screening method for identification of tolerant and sensitive mutants to low P condition in field. In our previous report, we identified EMS induced mutant of N22 which gave higher grain yield than N22 under high temperature^64,71^. One such EMS induced mutant of N22, ‘*ewst1*’ showed higher tolerance to water limited condition in terms of grain yield and the mutant differed from wild type in root traits, root anatomy and chalky endosperm^72^. 67 N22 mutants were screened in low P field, AWD and normal conditions and 6 of them NH787, NH686, NH669, NH363, NH355 and NH719 were found promising under these three conditions based on only wet season 2012 data^33^. Single marker analysis showed that 15 markers were associated with plant height, number of tillers, number of panicles and yield per plant and *Pup1* gene specific marker, K-1 showed significant association with tiller number at low P conditions. In our present expanded study of 12 seasons, only three of these NH787, NH686 and NH363 showed high yield and stability across the environments and seasons.

To our knowledge, this is the first attempt to study the yield stability of rice mutants across low P, water limited and normal irrigated conditions. MutMap method can be employed to identify the mutations causing stable stress tolerance. The genotypes that perform best in these environments may be used as reference genotypes in further abiotic stress breeding experiments and also explored to understand the genetic control of phenotypic plasticity leading to better yield stability^73^.

## Conclusions

This study led to identification of high yielding rice mutants which are suitable for irrigated as well as limited water and phosphorus conditions. EMS induced mutagenesis can lead to discovery of very useful mutants such as G17 (NH686) and G176 (NH363) which are stably high yielding across normal and input limited stresses. Two lines G17 (NH686) and G284 (NH162) showed better mean performance in water limited condition compared to normal irrigated condition while other mutants showed yield reduction. G17(NH686) was the most stable genotype with highest rank in both stability studies of 6 mutants in 3 environments for 12 seasons as well as 300 mutants in 3 environments for 1 season. The three high yielding stable mutants performed similarly in both genotype environment interaction studies. Both limited water and phosphorus resulted in reduction of average yield and biomass. However, the extent of reduction varied for each trait in each environment. Low G effect and high E effect was observed for grain yield and plant height, considering G × E analysis over 3 environments for 300 genotypes. Similarly selected lines tested over multiple years also showed high E effect for grain yield. However, G × E interaction was more significant in case of tiller number indicating the specific adaptation of this trait in stress and normal conditions. Environments representing specific conditions clustered together and GGE plot classified normal and low P stress conditions into two different mega environments while moderate stress of limited water condition clustered between these two mega environments. The stable and high yielding mutants in stress and normal conditions can be evaluated in larger plots in multiple locations and used in basic studies on gene discovery and in rice improvement for sustainable rice production.

## Electronic supplementary material

Supplementary Data set 1
