# Supplementary material for: Genotype × Environment interactions of Nagina22 rice mutants for yield traits under low phosphorus, water limited and normal irrigated conditions
Source: Sci Rep. 2018 Oct 19;8:15530. doi: 10.1038/s41598-018-33812-1 (PMC6195568; doi:10.1038/s41598-018-33812-1)
Supplement: Supplementary file 1 — Supplementary Data set 1 [file 41598_2018_33812_MOESM1_ESM.docx]

**Genotype** × **Environment interactions of Nagina22 rice mutants for yield traits under low phosphorus, water limited and normal irrigated conditions**

P. Yugandhar^1^, B. Divya^1^, D. Subrahmanyam^1^, M Panigrahy^1^, S. R. Voleti^1^, S. K. Mangrauthia^1^ and N. Sarla^1^*

**Supplementary Figures**

**
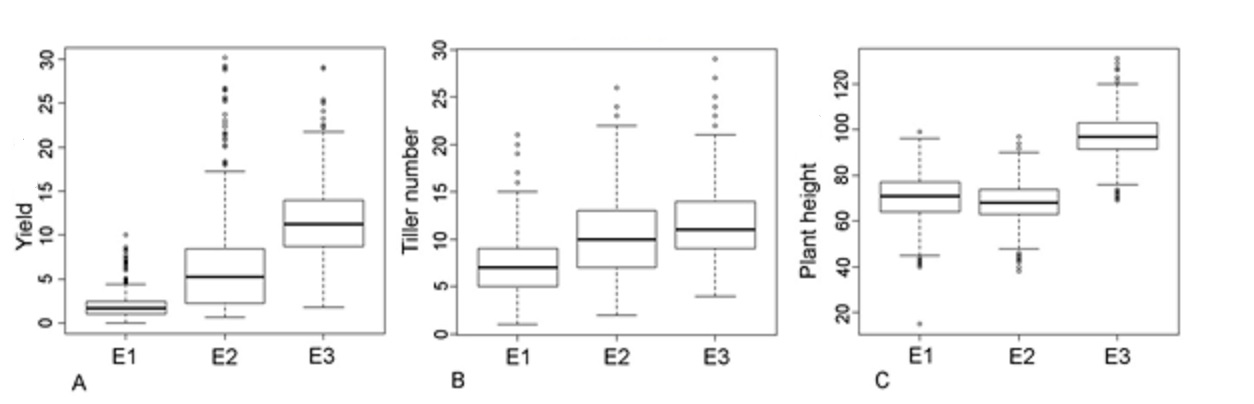
**

**Fig. S1.** Box plots in three environments. A) Yield (g/plant) B) Tiller number C) Plant height (cm)


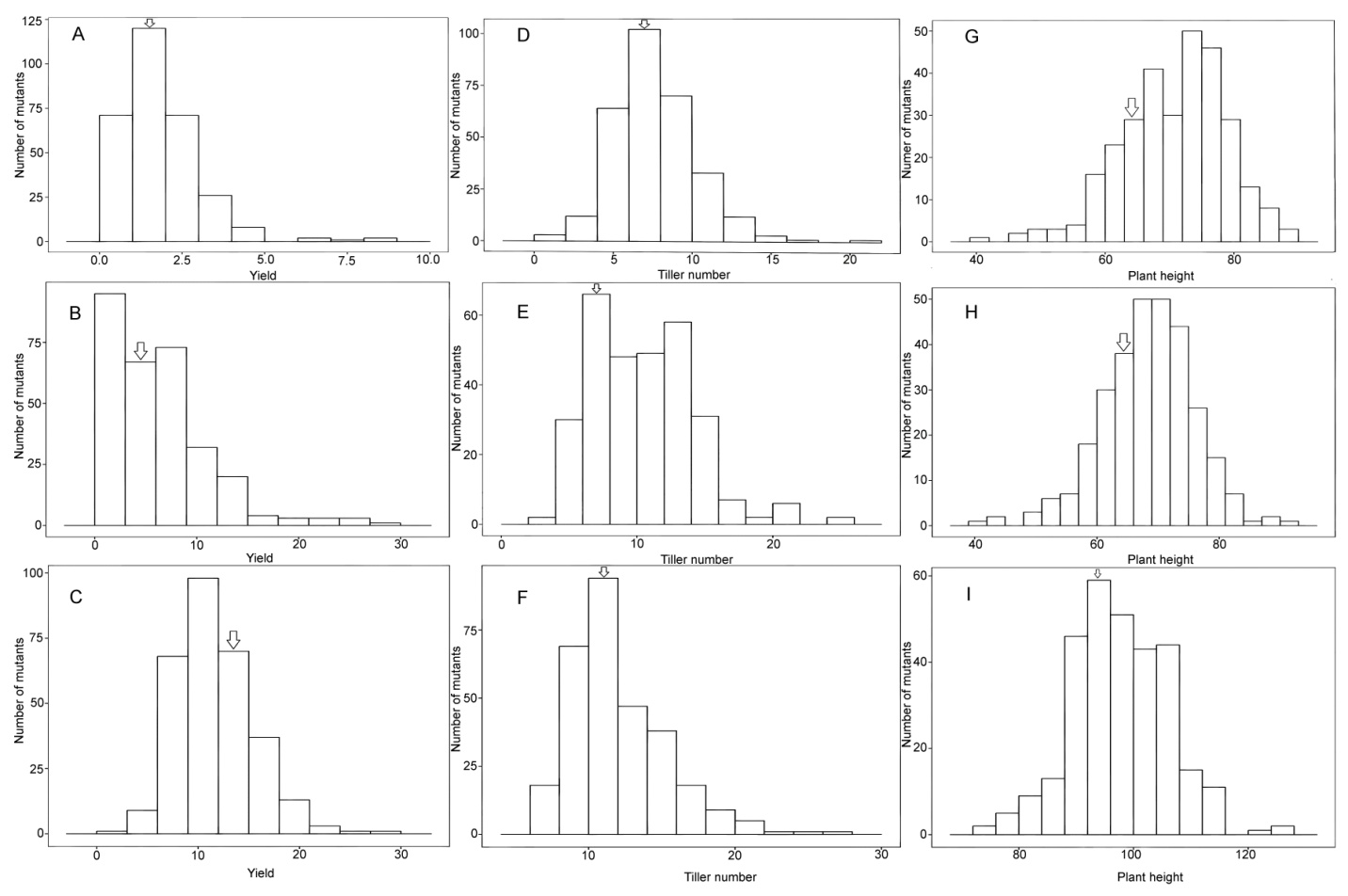


**Fig. S2.** Frequency distribution in three environments A) Yield in E1, B) Yield in E2, C) Yield in E3. D) Tiller number in E1, E) Tiller number in E2, F) Tiller number in E3. G) Plant height in E1, F) Plant height in E2, G) Plant height in E3. Arrow in each graph indicates N22

| 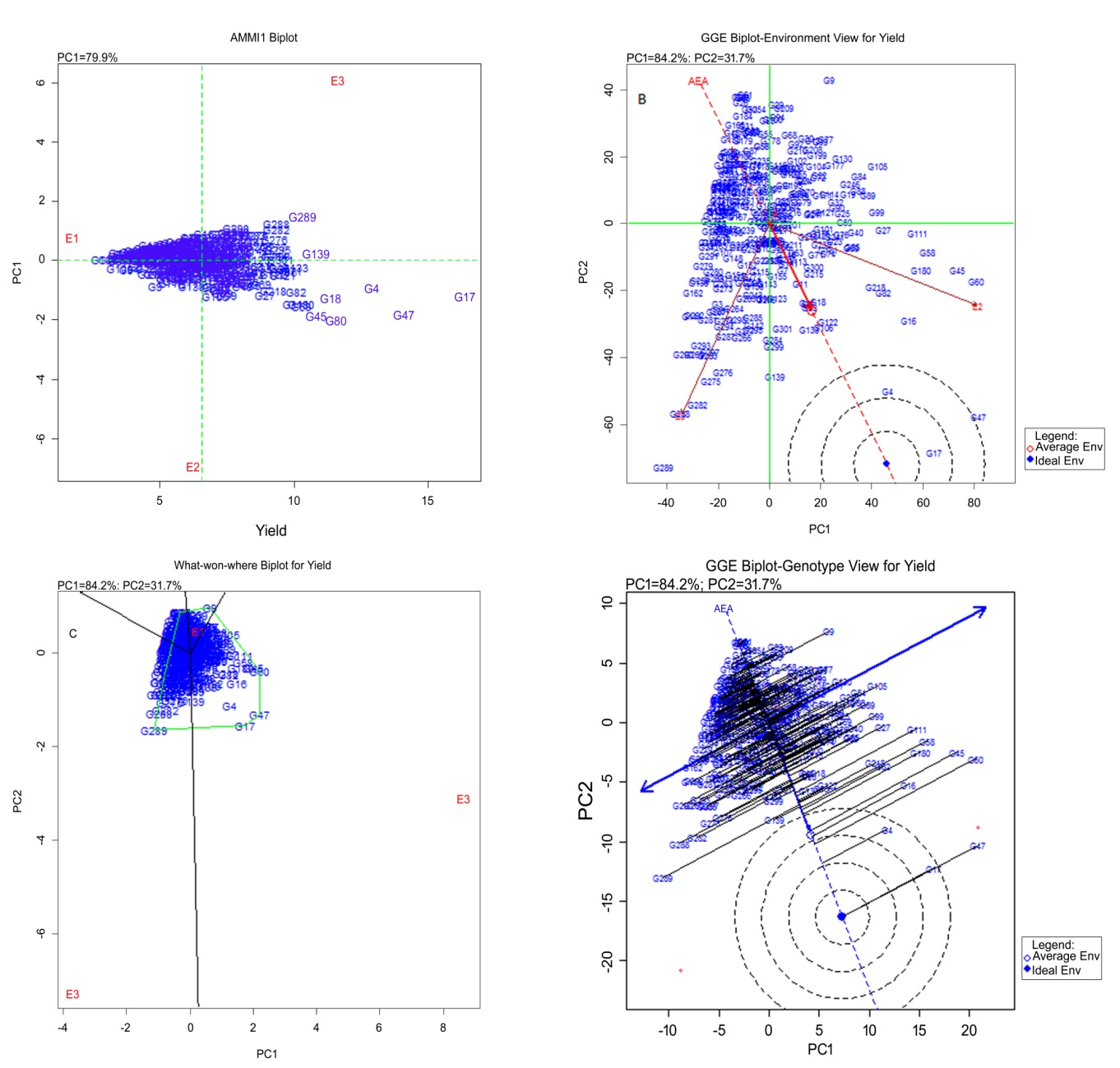 |
| --- |
| **Fig. S3.** AMMI1 biplots for grain yield (g / plant) of 301 mutants tested in three environments. B. GGE biplot of ideal genotype and comparison of the genotypes with ideal genotype. C. GGE biplot of 301 genotypes for grain yield in three environments based on which-won- where pattern. D. GGE biplot of genotype view for yield in comparison with ideal environment and average environment |
| 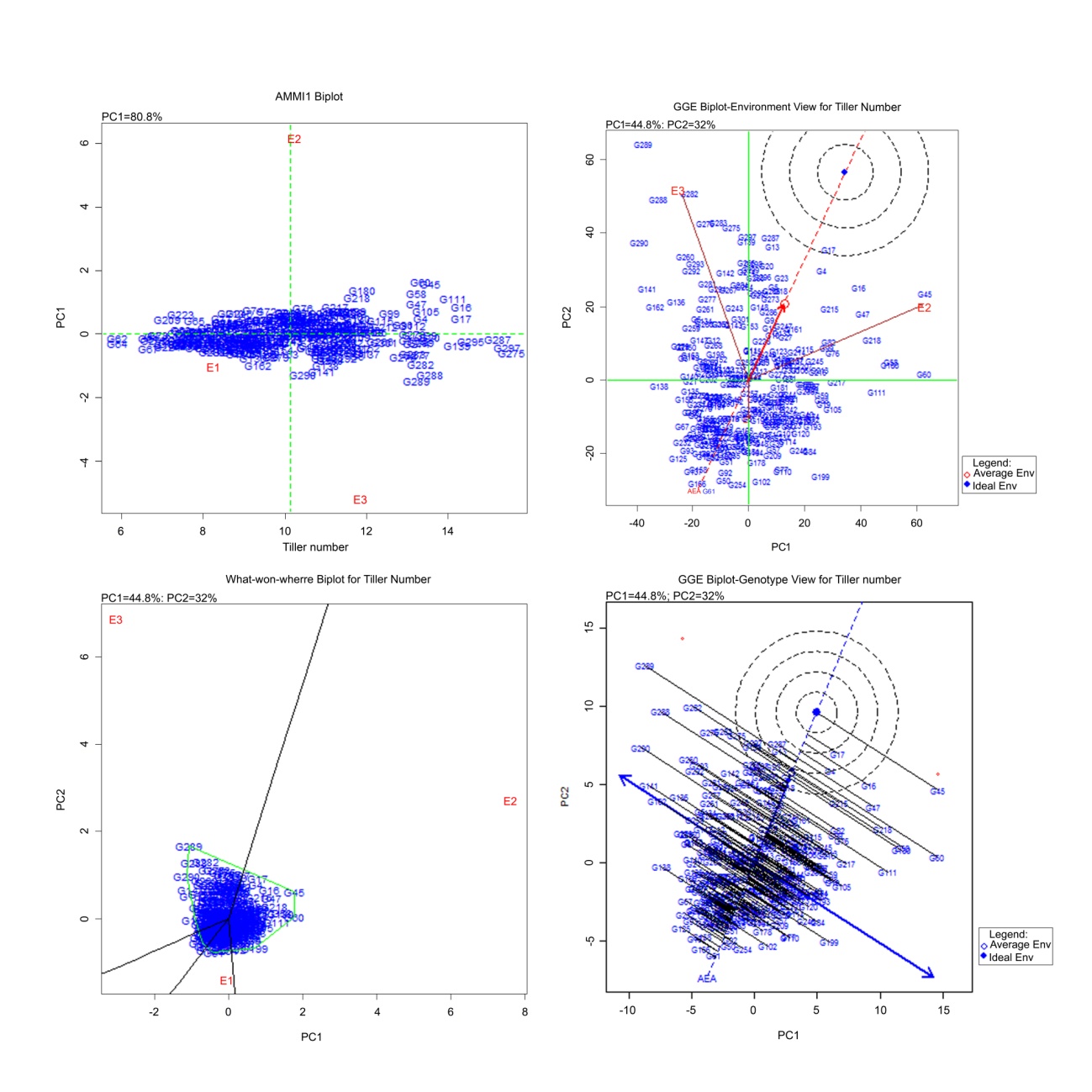 |
| **Fig. S4.** A. AMMI1 biplots for tiller number of 301 mutants tested in three environments. B. GGE biplot of ideal genotype and comparison of the genotypes with ideal genotype. C. GGE biplot of 301 genotypes for tiller number in three environments based on which-won- where pattern. D. GGE biplot of genotype view for tiller number in comparison with ideal environment and average environment |
| 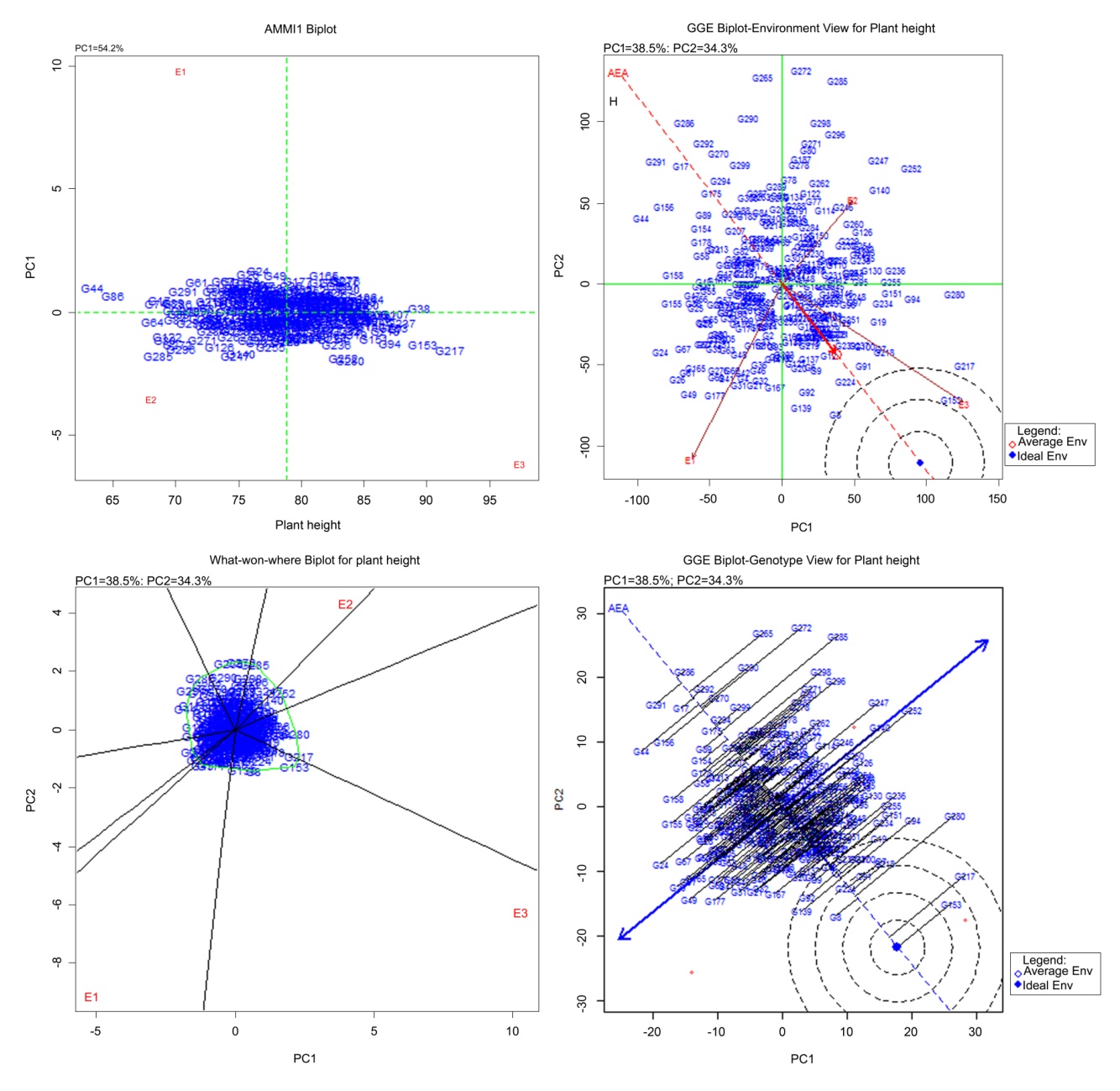 |
| **Fig. S5.** A. AMMI1 biplots for plant height of 301 mutants tested in three environments. B. GGE biplot of ideal genotype and comparison of the genotypes with ideal genotype. C. GGE biplot of 301 genotypes for plant height in three environments based on which-won- where pattern. D. GGE biplot of genotype view for plant height in comparison with ideal environment and average environment |


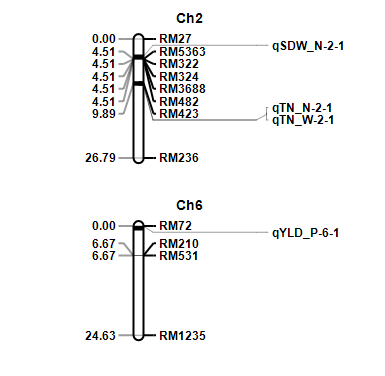


**Fig. S6.** Linkage map and marker trait association

**Supplementary Tables**

**Supplementary table 1**. List of genotypes and environments and the codes used in AMMI and GGE analysis

| **Code** | **Mutant Name** | **Code** | **Mutant Name** | **Code** | **Mutant Name** | **Code** | **Mutant Name** | **Code** | **Mutant Name** | **Code** | **Mutant Name** | **Code** | **Mutant Name** | **Code** | **Mutant Name** | **Code** | **Environment** |
| --- | --- | --- | --- | --- | --- | --- | --- | --- | --- | --- | --- | --- | --- | --- | --- | --- | --- |
| G1 | N22 | G41 | NH580 | G81 | NH493 | G121 | NH185 | G161 | NH443 | G201 | NH212 | G241 | NH254 | G281 | NH163 | **E1** | **Low P** |
| G2 | NH753 | G42 | NH655 | G82 | NH547 | G122 | NH519 | G162 | NH370 | G202 | NH184 | G242 | NH312 | G282 | NH214 | E1a | Rb09 |
| G3 | NH577 | G43 | NH737 | G83 | NH429 | G123 | NH209 | G163 | NH454 | G203 | NH86 | G243 | NH311 | G283 | NH157 | E1b | Rb10 |
| G4 | NH779 | G44 | NH646 | G84 | NH544 | G124 | NH253 | G164 | NH456 | G204 | NH89 | G244 | NH331 | G284 | NH162 | E1c | Kh11 |
| G5 | NH699 | G45 | NH783 | G85 | NH546 | G125 | NH210 | G165 | NH436 | G205 | NH211 | G245 | NH329 | G285 | NH146 | E1d | Rb11 |
| G6 | NH710 | G46 | NH675 | G86 | NH444 | G126 | NH177 | G166 | NH374 | G206 | NH90 | G246 | NH323 | G286 | NH138 | E1e | Kh12 |
| G7 | NH777 | G47 | NH677 | G87 | NH540 | G127 | NH182 | G167 | NH541 | G207 | NH186 | G247 | NH320 | G287 | NH85 | E1f | Rb12 |
| G8 | NH765 | G48 | NH673 | G88 | NH412 | G128 | NH180 | G168 | NH507 | G208 | NH310 | G248 | NH322 | G288 | NH172 | E1g | Kh13 |
| G9 | NH756 | G49 | NH743 | G89 | NH428 | G129 | NH91 | G169 | NH458 | G209 | NH351 | G249 | NH228 | G289 | NH23 | E1h | Rb13 |
| G10 | NH757 | G50 | NH681 | G90 | NH542 | G130 | NH178 | G170 | NH461 | G210 | NH241 | G250 | NH229 | G290 | NH137 | E1i | Kh14 |
| G11 | NH766 | G51 | NH729 | G91 | NH506 | G131 | NH185 | G171 | NH418 | G211 | NH259 | G251 | NH323 | G291 | NH110 | E1j | Rb14 |
| G12 | NH636 | G52 | NH738 | G92 | NH424 | G132 | NH176 | G172 | NH409 | G212 | NH52 | G252 | NH324 | G292 | NH115 | E1k | Kh15 |
| G13 | NH662 | G53 | NH761 | G93 | NH423 | G133 | NH213 | G173 | NH359 | G213 | NH50 | G253 | NH307 | G293 | NH20 | E1l | Rb15 |
| G14 | NH551 | G54 | NH556 | G94 | NH543 | G134 | NH181 | G174 | NH439 | G214 | NH97 | G254 | NH233 | G294 | NH45 | **E2** | **Water limited** |
| G15 | NH648 | G55 | NH558 | G95 | NH410 | G135 | NH501 | G175 | NH415 | G215 | NH56 | G255 | NH319 | G295 | NH175 | E2a | Rb09 |
| G16 | NH697 | G56 | NH549 | G96 | NH419 | G136 | NH299 | G176 | NH363 | G216 | NH26 | G256 | NH314 | G296 | NH41 | E2b | Rb10 |
| G17 | NH686 | G57 | NH575 | G97 | NH380 | G137 | NH60 | G177 | NH347 | G217 | NH111 | G257 | NH318 | G297 | NH84 | E2c | Kh11 |
| G18 | NH687 | G58 | NH651 | G98 | NH360 | G138 | NH61 | G178 | NH357 | G218 | NH109 | G258 | NH223 | G298 | NH33 | E2d | Rb11 |
| G19 | NH649 | G59 | NH553 | G99 | NH427 | G139 | NH48 | G179 | NH426 | G219 | NH122 | G259 | NH224 | G299 | NH25 | E2e | Kh12 |
| G20 | NH552 | G60 | NH698 | G100 | NH361 | G140 | NH47 | G180 | NH381 | G220 | NH35 | G260 | NH244 | G300 | NH170 | E2f | Rb12 |
| G21 | NH772 | G61 | NH683 | G101 | NH425 | G141 | NH28 | G181 | NH341 | G221 | NH563 | G261 | NH226 |  |  | E2g | Kh13 |
| G22 | NH650 | G62 | NH701 | G102 | NH510 | G142 | NH113 | G182 | NH342 | G222 | NH376 | G262 | NH147 |  |  | E2h | Rb13 |
| G23 | NH709 | G63 | NH717 | G103 | NH346 | G143 | NH100 | G183 | NH293 | G223 | NH642 | G263 | NH18 |  |  | E2i | Kh14 |
| G24 | NH759 | G64 | NH695 | G104 | NH348 | G144 | NH107 | G184 | NH477 | G224 | NH572 | G264 | NH8 |  |  | E2j | Rb14 |
| G25 | NH574 | G65 | NH787 | G105 | NH355 | G145 | NH106 | G185 | NH362 | G225 | NH36 | G265 | NH222 |  |  | E2k | Kh15 |
| G26 | NH685 | G66 | NH744 | G106 | NH414 | G146 | NH645 | G186 | NH302 | G226 | NH30 | G266 | NH236 |  |  | E2l | Rb15 |
| G27 | NH652 | G67 | NH720 | G107 | NH368 | G147 | NH670 | G187 | NH263 | G227 | NH397 | G267 | NH220 |  |  | **E3** | **Normal** |
| G28 | NH727 | G68 | NH755 | G108 | NH460 | G148 | NH664 | G188 | NH356 | G228 | NH326 | G268 | NH240 |  |  | E3a | Rb09 |
| G29 | NH764 | G69 | NH579 | G109 | NH294 | G149 | NH676 | G189 | NH265 | G229 | NH327 | G269 | NH245 |  |  | E3b | Rb10 |
| G30 | NH671 | G70 | NH562 | G110 | NH276 | G150 | NH40 | G190 | NH413 | G230 | NH325 | G270 | NH238 |  |  | E3c | Kh11 |
| G31 | NH661 | G71 | NH767 | G111 | NH430 | G151 | NH462 | G191 | NH514 | G231 | NH357 | G271 | NH239 |  |  | E3d | Rb11 |
| G32 | NH637 | G72 | NH774 | G112 | NH474 | G152 | NH406 | G192 | NH517 | G232 | NH335 | G272 | NH237 |  |  | E3e | Kh12 |
| G33 | NH659 | G73 | NH690 | G113 | NH264 | G153 | NH403 | G193 | NH266 | G233 | NH334 | G273 | NH219 |  |  | E3f | Rb12 |
| G34 | NH639 | G74 | NH680 | G114 | NH192 | G154 | NH405 | G194 | NH504 | G234 | NH340 | G274 | NH152 |  |  | E3g | Kh13 |
| G35 | NH656 | G75 | NH647 | G115 | NH438 | G155 | NH404 | G195 | NH491 | G235 | NH399 | G275 | NH216 |  |  | E3h | Rb13 |
| G36 | NH704 | G76 | NH648 | G116 | NH376 | G156 | NH548 | G196 | NH500 | G236 | NH328 | G276 | NH218 |  |  | E3i | Kh14 |
| G37 | NH678 | G77 | NH408 | G117 | NH207 | G157 | NH502 | G197 | NH290 | G237 | NH390 | G277 | NH168 |  |  | E3j | Rb14 |
| G38 | NH778 | G78 | NH484 | G118 | NH270 | G158 | NH431 | G198 | NH503 | G238 | NH333 | G278 | NH215 |  |  | E3k | Kh15 |
| G39 | NH653 | G79 | NH479 | G119 | NH516 | G159 | NH434 | G199 | NH252 | G239 | NH332 | G279 | NH167 |  |  | E3l | Rb15 |
| G40 | NH682 | G80 | NH494 | G120 | NH282 | G160 | NH445 | G200 | NH249 | G240 | NH338 | G280 | NH247 |  |  |  |  |

NH- Nagina 22 Hyderabad mutants, G - genotype, E- environment, Kh- kharif wet season, Rb- rabi dry season

**Supplementary table 2. List of markers used for genotyping**

| **Sl. No.** | **Marker Name** | **Primer Sequence** | |
| --- | --- | --- | --- |
|  |  | **Forward primer** | **Reverse primer** |
| **Pup1 specific markers Heuer et al. (2009)** | | | |
| 1 | K1 | AGTCTGGATGGACAACTCTGCCTG | TGCTAGCTCATTGCCGTTACGTCG |
| 2 | K5 | ATTCAGACATCGACGGCGAC | TCCTCGTAAACATGGCTTGC |
| 3 | K20-1 | TCAGGTGATGGGAATCATTG | TGTTCCAACCAAACAACCTG |
| 4 | K29-1 | ATGGCCAACGGGGTAGAG | GTCCAGGTAACCACGAGGAA |
| 5 | K29-2 | CCCGTCTGCGTTCTACCTTA | CTCCCGTCAAGCACAAATCT |
| 6 | K29-3 | TTCGTCCAGATGCTGCTATG | TCTTCGGTGTAATTGGCACA |
| 7 | K41 | TGATGAATCCATAGGACAGCGT | TCAGGTGGTGCTTCGTTGGTA |
| 8 | K42 | CCCGAGAGTTCATCAGAAGGA | AGTGAGTGGCGTTTGCGAT |
| 9 | K43 | AGGAGGATGAGCCTGAAGAGA | TCGCACTAACAGCAGCAGATT |
| 10 | K45 | GCGGAAGAAGAGGATAACGA | TCTAGGCTTCGTTTGGCAAG |
| 11 | K48 | CAGCATTCAGCAAGACAACAG | ATCCGTGTGGAGCAACTCATC |
| 12 | K52 | ACCGTTCCCAACAGATTCCAT | CCCGTAATAGCAACAACCCAA |
| 13 | K46-1 | TGAGATAGCCGTCAAGATGCT | AAGGACCACCATTCCATAGC |
| 14 | K46-2 | AGGAAGATGGTTGTCGTTGG | TTCACACCAAACAGTGTTGTC |
| 15 | K48 | CAGCATTCAGCAAGACAACAG | ATCCGTGTGGAGCAACTCATC |
| **SSR markers** | | | |
| 1 | RM322 | CAAGCGAAAATCCCAGCAG | CAAGCGAAAATCCCAGCAG |
| 2 | RM291 | GTTGCACTACGTATTCTGAG | GTTGCACTACGTATTCTGAG |
| 3 | RM316 | CTAGTTGGGCATACGATGGC | ACGCTTATATGTTACGTCAAC |
| 4 | RM247 | TAGTGCCGATCGATGTAACG | CATATGGTTTTGACAAAGCG |
| 5 | RM235 | AGAAGCTAGGGCTAACGAAC | TCACCTGGTCAGCCTCTTTC |
| 6 | RM297 | TCTTTGGAGGCGAGCTGAG | CGAAGGGTACATCTGCTTAG |
| 7 | RM322 | CAAGCGAAAATCCCAGCAG | GATGAAACTGGCATTGCCTG |
| 8 | RM302 | TCATGTCATCTACCATCACAC | ATGGAGAAGATGGAATACTTGC |
| 9 | RM322 | CAAGCGAAAATCCCAGCAG | GATGAAACTGGCATTGCCTG |
| 10 | RM291 | GTTGCACTACGTATTCTGAG | GATCCAGATAAATGAGGCAC |
| 11 | RM247 | TAGTGCCGATCGATGTAACG | CATATGGTTTTGACAAAGCG |
| 12 | RM72 | CCGGCGATAAAACAATGAG | GCATCGGTCCTAACTAAGGG |
| 13 | RM160 | AGCTAGCAGCTATAGCTTAGCTGGAGATC | TCTCATCGCCATGCGAGGCCTC |
| 14 | RM219 | CGTCGGATGATGTAAAGCCT | CATATCGGCATTCGCCTG |
| 15 | RM3790 | TAATTGCGGTCTCGTGCC | AACCACCTCAACTACTGCCG |
| 16 | RM215 | CAAAATGGAGCAGCAAGAGC | TGAGCACCTCCTTCTCTGTAG |
| 17 | RM212 | CCACTTTCAGCTACTACCAG | CACCCATTTGTCTCTCATTATG |
| 18 | RM302 | TCATGTCATCTACCATCACAC | ATGGAGAAGATGGAATACTTGC |
| 19 | RM3825 | AAAGCCCCCAAAAGCAGTAC | GTGAAACTCTGGGGTGTTCG |
| 20 | RM212 | CCACTTTCAGCTACTACCAG | CACCCATTTGTCTCTCATTATG |
| 21 | RM1235 | AGCAGAGGAGGAGATGATGG | GGACCAAAACGAAGCTATCC |
| 22 | RM6862 | GGCAAGATCGTTGGAAGAAC | TTACCTGTCGTTTCCCTTCG |
| 23 | RM6449 | CAAGAGCAGCAGCTTGACAG | GCGTAGGGACTAGGAGAGGG |
| 24 | RM1859 | TCGTAAGAACATGGAGAACC | GGATTTTCTGATAGCGGTAA |
| 25 | RM304 | TCAAACCGGCACATATAAGAC | GATAGGGAGCTGAAGGAGATG |
| 26 | RM236 | GCGCTGGTGGAAAATGAG | GGCATCCCTCTTTGATTCCTC |
| 27 | RM423 | AGCACCCATGCCTTATGTTG | CCTTTTTCAGTAGCCCTCCC |
| 28 | RM531 | GAAACATCCCATGTTCCCAC | TCGGTTTTTCAGACTCGGTC |
| 29 | RM591 | CTAGCTAGCTGGCACCAGTG | TGGAGTCCGTGTTGTAGTCG |
| 30 | RM3366 | TGTTTTGCGTATTTATAGGATG | CAAGAAGTACATGGGACCTG |
| 31 | RM273 | GAAGCCGTCGTGAAGTTACC | GTTTCCTACCTGATCGCGAC |
| 32 | RM553 | AACTCCACATGATTCCACCC | GAGAAGGTGGTTGCAGAAGC |
| 33 | RM538 | GGTCGTTGAAGCTTACCAGC | ACAAGCTCTCAAAACTCGCC |
| 34 | RM584 | AGAAAGTGGATCAGGAAGGC | GATCCTGCAGGTAACCACAC |
| 35 | RM1089 | CAGAAGGATTATCTCGATACC | AATAGGGCTTGAAATAAATTG |
| 36 | RM8007 | AATAGGATGGATCATGGATA | CATCTCATCAGGAACCTAAC |
| 37 | RM448 | TCTGATCTTGATGCAGGCAC | TCTCCCGATTTGGACAGATC |
| 38 | RM551 | AGCCCAGACTAGCATGATTG | GAAGGCGAGAAGGATCACAG |
| 39 | RM5363 | TCCCTCCCTGGCTTTTTTAC | AGCAACGCGGTGAGAGAC |
| 40 | RM434 | GCCTCATCCCTCTAACCCTC | CAAGAAAGATCAGTGCGTGG |
| 41 | RM453 | CGCATCTCTCTCCCTTATCG | CTCTCCTCCTCGTTGTCGTC |
| 42 | RM3688 | GTTGAATCAAGCTGTGCAGC | AGCTAGGCAAAGCATGCATG |
| 43 | RM210 | TCACATTCGGTGGCATTG | CGAGGATGGTTGTTCACTTG |
| 44 | RM5363 | TCCCTCCCTGGCTTTTTTAC | AGCAACGCGGTGAGAGAC |
| 45 | RM434 | GCCTCATCCCTCTAACCCTC | CAAGAAAGATCAGTGCGTGG |
| 46 | RM448 | TCTGATCTTGATGCAGGCAC | TCTCCCGATTTGGACAGATC |
| 47 | RM511 | CTTCGATCCGGTGACGAC | AACGAAAGCGAAGCTGTCTC |
| 48 | RM324 | CTGATTCCACACACTTGTGC | GATTCCACGTCAGGATCTTC |
| 49 | RM27 | TTTTCCTTCTCACCCACTTCA | TCTTTGACAAGAGGAAAGAGGC |
| 50 | RM482 | TCTGAAAGCCTGACTCATCG | GTCAATTGCAGTGCCCTTTC |

**Supplementary table 3.A.** Analysis of variance of grain yield in 6 mutants and N22 in low- P condition for 12 seasons

|  | d.f. | Sum of Squares | Mean Squares | F | p.value | SS% |
| --- | --- | --- | --- | --- | --- | --- |
| Total | 83 | 2063.71 |  |  |  |  |
| Genotypes | 6 | 2053.37 | 342.23 | 3872.38 | <0.001 | 99.50 |
| Environments | 11 | 4.51 | 0.41 | 8.32 | <0.001 | 0.22 |
| Interaction | 66 | 5.83 | 0.09 | 1.79 | 0.00 | 0.28 |
| Heterogeneity | 6 | 3.55 | 0.59 | 15.52 | <0.001 | 0.17 |
| Residual | 60 | 2.29 | 0.04 | 0.77 | 0.87 | 0.11 |
| Pooled error | 144 |  | 0.05 |  |  |  |

**Supplementary table 3. B.** Analysis of variance of grain yield in 6 mutants and N22 in water limited condition 12 seasons

|  | d.f. | Sum of Squares | Mean Squares | F | p.value | SS% |
| --- | --- | --- | --- | --- | --- | --- |
| Total | 83 | 15442.23 |  |  |  |  |
| Genotypes | 6 | 15000.61 | 500.10 | 639.59 | <0.001 | 97.14 |
| Environments | 11 | 183.63 | 16.69 | 43.36 | <0.001 | 1.19 |
| Interaction | 66 | 257.99 | 3.91 | 10.15 | <0.001 | 1.67 |
| Heterogeneity | 6 | 42.74 | 7.12 | 1.99 | 0.08 | 0.28 |
| Residual | 60 | 215.25 | 3.59 | 9.32 | <0.001 | 1.39 |
| Pooled error | 144 |  | 0.385 |  |  |  |

**Supplementary table 3. C.** Analysis of variance of grain yield in 6 mutants and N22 in normal condition 12 seasons

|  | d.f. | Sum of Squares | Mean Squares | F | p.value | SS% |
| --- | --- | --- | --- | --- | --- | --- |
| Total | 83 | 1537.52 |  |  |  |  |
| Genotypes | 6 | 998.68 | 166.45 | 152.71 | <0.001 | 64.95 |
| Environments | 11 | 466.91 | 42.45 | 235.81 | <0.001 | 30.37 |
| Interaction | 66 | 71.94 | 1.09 | 6.06 | <0.001 | 4.68 |
| Heterogeneity | 6 | 3.40 | 0.57 | 0.50 | 0.81 | 0.22 |
| Residual | 60 | 68.53 | 1.14 | 6.35 | <0.001 | 4.46 |
| Pooled error | 144 |  | 0.18 |  |  |  |

**Supplementary table 3. D.** Analysis of variance of grain yield in 6 mutants and N22 in low-P, water limited and normal conditions

|  | d.f. | Sum of Squares | Mean Squares | F | p.value | SS% |
| --- | --- | --- | --- | --- | --- | --- |
| Total | 251 | 38390.58 |  |  |  |  |
| Genotypes | 6 | 9459.11 | 1576.52 | 37.08 | <0.001 | 24.64 |
| Environments | 35 | 20002.17 | 571.49 | 2811.07 | <0.001 | 52.10 |
| Interaction | 210 | 8929.30 | 42.52 | 209.15 | <0.001 | 23.26 |
| Heterogeneity | 6 | 1009.05 | 168.18 | 4.33 | <0.001 | 2.63 |
| Residual | 204 | 7920.25 | 38.82 | 190.97 | <0.001 | 20.63 |
| Pooled error | 432 |  | 0.20 |  |  |  |

**Supplementary Table 4A.** YSi statistic for grain yield / plant in 300 mutants

| Code | Mutant | YSi (Y+S) | Superior mutants (+) | Code | Mutant | YSi (Y+S) | Superior mutants (+) | Code | Mutant | YSi (Y+S) | Superior mutants (+) |
| --- | --- | --- | --- | --- | --- | --- | --- | --- | --- | --- | --- |
| G1 | N22 | 143 |  | G47 | NH677 | 295 | + | G93 | NH423 | 24 |  |
| G2 | NH753 | 98 |  | G48 | NH673 | 8 |  | G94 | NH543 | 5 |  |
| G3 | NH577 | 197 | + | G49 | NH743 | -9 |  | G95 | NH410 | 195 | + |
| G4 | NH779 | 294 | + | G50 | NH681 | 12 |  | G96 | NH419 | 222 | + |
| G5 | NH699 | 268 | + | G51 | NH729 | 201 | + | G97 | NH380 | 196 | + |
| G6 | NH710 | 223 | + | G52 | NH738 | 19 |  | G98 | NH360 | 136 |  |
| G7 | NH777 | 241 | + | G53 | NH761 | 20 |  | G99 | NH427 | 218 | + |
| G8 | NH765 | 88 |  | G54 | NH556 | 221 | + | G100 | NH361 | -5 |  |
| G9 | NH756 | 39 |  | G55 | NH558 | 50 |  | G101 | NH425 | 180 | + |
| G10 | NH757 | 198 | + | G56 | NH549 | 34 |  | G102 | NH510 | 46 |  |
| G11 | NH766 | 266 | + | G57 | NH575 | 38 |  | G103 | NH346 | 35 |  |
| G12 | NH636 | 97 |  | G58 | NH651 | 288 | + | G104 | NH348 | 71 |  |
| G13 | NH662 | 62 |  | G59 | NH553 | 249 | + | G105 | NH355 | 189 | + |
| G14 | NH551 | 164 | + | G60 | NH698 | 293 | + | G106 | NH414 | 284 | + |
| G15 | NH648 | 223 | + | G61 | NH683 | -8 |  | G107 | NH368 | 204 | + |
| G16 | NH697 | 292 | + | G62 | NH701 | 22 |  | G108 | NH460 | 68 |  |
| G17 | NH686 | 296 | + | G63 | NH717 | 258 | + | G109 | NH294 | 108 |  |
| G18 | NH687 | 262 | + | G64 | NH695 | 152 | + | G110 | NH276 | 103 |  |
| G19 | NH649 | 183 | + | G65 | NH787 | 157 | + | G111 | NH430 | 286 | + |
| G20 | NH552 | 281 | + | G66 | NH744 | 176 | + | G112 | NH474 | 45 |  |
| G21 | NH772 | 111 |  | G67 | NH720 | 55 |  | G113 | NH264 | 204 | + |
| G22 | NH650 | 118 |  | G68 | NH755 | 72 |  | G114 | NH192 | 178 | + |
| G23 | NH709 | 217 | + | G69 | NH579 | 265 | + | G115 | NH438 | 213 | + |
| G24 | NH759 | 116 |  | G70 | NH562 | 170 | + | G116 | NH376 | 110 |  |
| G25 | NH574 | 231 | + | G71 | NH767 | 163 | + | G117 | NH207 | 14 |  |
| G26 | NH685 | -1 |  | G72 | NH774 | 172 | + | G118 | NH270 | 66 |  |
| G27 | NH652 | 269 | + | G73 | NH690 | 0 |  | G119 | NH516 | 138 |  |
| G28 | NH727 | -6 |  | G74 | NH680 | 259 | + | G120 | NH282 | 74 |  |
| G29 | NH764 | 21 |  | G75 | NH647 | 86 |  | G121 | NH185 | 283 | + |
| G30 | NH671 | 102 |  | G76 | NH648 | 255 | + | G122 | NH519 | 273 | + |
| G31 | NH661 | 9 |  | G77 | NH408 | 68 |  | G123 | NH209 | 263 | + |
| G32 | NH637 | 229 | + | G78 | NH484 | 16 |  | G124 | NH253 | 207 | + |
| G33 | NH659 | 28 |  | G79 | NH479 | 106 |  | G125 | NH210 | 31 |  |
| G34 | NH639 | 80 |  | G80 | NH494 | 123 |  | G126 | NH177 | 234 | + |
| G35 | NH656 | -4 |  | G81 | NH493 | 85 |  | G127 | NH182 | 49 |  |
| G36 | NH704 | 108 |  | G82 | NH547 | 287 | + | G128 | NH180 | 81 |  |
| G37 | NH678 | -3 |  | G83 | NH429 | 164 | + | G129 | NH91 | 70 |  |
| G38 | NH778 | 3 |  | G84 | NH544 | 194 | + | G130 | NH178 | 121 |  |
| G39 | NH653 | 77 |  | G85 | NH546 | 256 | + | G131 | NH185 | 64 |  |
| G40 | NH682 | 250 | + | G86 | NH444 | 251 | + | G132 | NH176 | 200 | + |
| G41 | NH580 | 18 |  | G87 | NH540 | 15 |  | G133 | NH213 | 285 | + |
| G42 | NH655 | 128 |  | G88 | NH412 | 202 | + | G134 | NH181 | 174 | + |
| G43 | NH737 | 156 | + | G89 | NH428 | 209 | + | G135 | NH501 | 65 |  |
| G44 | NH646 | 280 | + | G90 | NH542 | 196 | + | G136 | NH299 | 179 | + |
| G45 | NH783 | 291 | + | G91 | NH506 | 167 | + | G137 | NH60 | 36 |  |
| G46 | NH675 | 7 |  | G92 | NH424 | 63 |  | G138 | NH61 | 134 |  |
| G139 | NH48 | 290 | + | G186 | NH302 | 32 |  | G233 | NH334 | 40 |  |
| G140 | NH47 | 105 |  | G187 | NH263 | 117 |  | G234 | NH340 | 130 |  |
| G141 | NH28 | 225 | + | G188 | NH356 | 130 |  | G235 | NH399 | 41 |  |
| G142 | NH113 | 260 | + | G189 | NH265 | 16 |  | G236 | NH328 | 33 |  |
| G143 | NH100 | 242 | + | G190 | NH413 | 43 |  | G237 | NH390 | 236 | + |
| G144 | NH107 | 76 |  | G191 | NH514 | 114 |  | G238 | NH333 | 248 | + |
| G145 | NH106 | 245 | + | G192 | NH517 | 73 |  | G239 | NH332 | 140 |  |
| G146 | NH645 | 61 |  | G193 | NH266 | 98 |  | G240 | NH338 | 13 |  |
| G147 | NH670 | 139 |  | G194 | NH504 | 126 |  | G241 | NH254 | 230 | + |
| G148 | NH664 | 162 | + | G195 | NH491 | 185 | + | G242 | NH312 | 218 | + |
| G149 | NH676 | 84 |  | G196 | NH500 | 75 |  | G243 | NH311 | 227 | + |
| G150 | NH40 | 91 |  | G197 | NH290 | 36 |  | G244 | NH331 | 160 | + |
| G151 | NH462 | 155 | + | G198 | NH503 | 120 |  | G245 | NH329 | 140 |  |
| G152 | NH406 | 153 | + | G199 | NH252 | 112 |  | G246 | NH323 | 186 | + |
| G153 | NH403 | 191 | + | G200 | NH249 | 133 |  | G247 | NH320 | 83 |  |
| G154 | NH405 | 58 |  | G201 | NH212 | 182 | + | G248 | NH322 | 62 |  |
| G155 | NH404 | 206 | + | G202 | NH184 | 90 |  | G249 | NH228 | 142 |  |
| G156 | NH548 | 99 |  | G203 | NH86 | 184 | + | G250 | NH229 | 154 | + |
| G157 | NH502 | 78 |  | G204 | NH89 | 52 |  | G251 | NH323 | 145 | + |
| G158 | NH431 | 1 |  | G205 | NH211 | 89 |  | G252 | NH324 | 111 |  |
| G159 | NH434 | 158 | + | G206 | NH90 | 216 | + | G253 | NH307 | 179 | + |
| G160 | NH445 | 141 |  | G207 | NH186 | 147 | + | G254 | NH233 | 64 |  |
| G161 | NH443 | 244 | + | G208 | NH310 | 96 |  | G255 | NH319 | 11 |  |
| G162 | NH370 | 169 | + | G209 | NH351 | 57 |  | G256 | NH314 | 67 |  |
| G163 | NH454 | 124 |  | G210 | NH241 | 26 |  | G257 | NH318 | 114 |  |
| G164 | NH456 | 87 |  | G211 | NH259 | 192 | + | G258 | NH223 | 183 | + |
| G165 | NH436 | 157 | + | G212 | NH52 | 213 | + | G259 | NH224 | 130 |  |
| G166 | NH374 | -3 |  | G213 | NH50 | 150 | + | G260 | NH244 | 235 | + |
| G167 | NH541 | 95 |  | G214 | NH97 | 160 | + | G261 | NH226 | 214 | + |
| G168 | NH507 | 115 |  | G215 | NH56 | 253 | + | G262 | NH147 | 92 |  |
| G169 | NH458 | 29 |  | G216 | NH26 | 193 | + | G263 | NH18 | 161 | + |
| G170 | NH461 | 6 |  | G217 | NH111 | 208 | + | G264 | NH8 | 243 | + |
| G171 | NH418 | 123 |  | G218 | NH109 | 274 | + | G265 | NH222 | 228 | + |
| G172 | NH409 | 67 |  | G219 | NH122 | 181 | + | G266 | NH236 | 264 | + |
| G173 | NH359 | 51 |  | G220 | NH35 | 43 |  | G267 | NH220 | 187 | + |
| G174 | NH439 | 183 | + | G221 | NH563 | 79 |  | G268 | NH240 | 129 |  |
| G175 | NH415 | 32 |  | G222 | NH376 | 198 | + | G269 | NH245 | 101 |  |
| G176 | NH363 | 233 | + | G223 | NH642 | 82 |  | G270 | NH238 | 23 |  |
| G177 | NH347 | 126 |  | G224 | NH572 | 47 |  | G271 | NH239 | 169 | + |
| G178 | NH357 | 52 |  | G225 | NH36 | 10 |  | G272 | NH237 | 177 | + |
| G179 | NH426 | 6 |  | G226 | NH30 | 35 |  | G273 | NH219 | 199 | + |
| G180 | NH381 | 288 | + | G227 | NH397 | 147 | + | G274 | NH152 | 254 | + |
| G181 | NH341 | 220 | + | G228 | NH326 | 166 | + | G275 | NH216 | 261 | + |
| G182 | NH342 | 29 |  | G229 | NH327 | 211 | + | G276 | NH218 | 270 | + |
| G183 | NH293 | 4 |  | G230 | NH325 | 125 |  | G277 | NH168 | 272 | + |
| G184 | NH477 | -10 |  | G231 | NH357 | 59 |  | G278 | NH215 | 154 | + |
| G185 | NH362 | -2 |  | G232 | NH335 | 42 |  | G279 | NH167 | 133 |  |
| G280 | NH247 | 137 |  |  |  |  |  |  |  |  |  |
| G281 | NH163 | 224 | + |  |  |  |  |  |  |  |  |
| G282 | NH214 | 276 | + |  |  |  |  |  |  |  |  |
| G283 | NH157 | 267 | + |  |  |  |  |  |  |  |  |
| G284 | NH162 | 275 | + |  |  |  |  |  |  |  |  |
| G285 | NH146 | 240 | + |  |  |  |  |  |  |  |  |
| G286 | NH138 | 247 | + |  |  |  |  |  |  |  |  |
| G287 | NH85 | 257 | + |  |  |  |  |  |  |  |  |
| G288 | NH172 | 277 | + |  |  |  |  |  |  |  |  |
| G289 | NH23 | 271 | + |  |  |  |  |  |  |  |  |
| G290 | NH137 | 234 | + |  |  |  |  |  |  |  |  |
| G291 | NH110 | 113 |  |  |  |  |  |  |  |  |  |
| G292 | NH115 | 209 | + |  |  |  |  |  |  |  |  |
| G293 | NH20 | 237 | + |  |  |  |  |  |  |  |  |
| G294 | NH45 | 246 | + |  |  |  |  |  |  |  |  |
| G295 | NH175 | 279 | + |  |  |  |  |  |  |  |  |
| G296 | NH41 | 181 | + |  |  |  |  |  |  |  |  |
| G297 | NH84 | 236 | + |  |  |  |  |  |  |  |  |
| G298 | NH33 | 245 | + |  |  |  |  |  |  |  |  |
| G299 | NH25 | 277 | + |  |  |  |  |  |  |  |  |
| G300 | NH170 | 251 | + |  |  |  |  |  |  |  |  |
| G301 | NH123 | 282 | + |  |  |  |  |  |  |  |  |

Superior Mutants are lines with higher value for the trait with significant stability

| Yield Mean: 6.56 |
| --- |
| YS Mean: 143.71 |
| LSD (0.05): 0.78 |

**Supplementary Table 4B.** YSi statistic for tiller number / plant in 300 mutants

| Code | Mutant | YSi (Y+S) | Superior mutants (+) | Code | Mutant | YSi (Y+S) | Superior mutants (+) | Code | Mutant | YSi (Y+S) | Superior mutants (+) |
| --- | --- | --- | --- | --- | --- | --- | --- | --- | --- | --- | --- |
| G1 | N22 | 97 |  | G47 | NH677 | 281 | + | G93 | NH423 | 47 |  |
| G2 | NH753 | 160 | + | G48 | NH673 | 35 |  | G94 | NH543 | 60 |  |
| G3 | NH577 | 135 |  | G49 | NH743 | 73 |  | G95 | NH410 | 161 | + |
| G4 | NH779 | 284 | + | G50 | NH681 | -2 |  | G96 | NH419 | 43 |  |
| G5 | NH699 | 266 | + | G51 | NH729 | 89 |  | G97 | NH380 | 162 | + |
| G6 | NH710 | 229 | + | G52 | NH738 | 52 |  | G98 | NH360 | 196 | + |
| G7 | NH777 | 256 | + | G53 | NH761 | 26 |  | G99 | NH427 | 271 | + |
| G8 | NH765 | 109 |  | G54 | NH556 | 109 |  | G100 | NH361 | 23 |  |
| G9 | NH756 | 281 | + | G55 | NH558 | 43 |  | G101 | NH425 | 181 | + |
| G10 | NH757 | 237 | + | G56 | NH549 | 49 |  | G102 | NH510 | 26 |  |
| G11 | NH766 | 189 | + | G57 | NH575 | 109 |  | G103 | NH346 | 98 |  |
| G12 | NH636 | 181 | + | G58 | NH651 | 281 | + | G104 | NH348 | 157 | + |
| G13 | NH662 | 278 | + | G59 | NH553 | 176 | + | G105 | NH355 | 272 | + |
| G14 | NH551 | 169 | + | G60 | NH698 | 284 | + | G106 | NH414 | 204 | + |
| G15 | NH648 | 245 | + | G61 | NH683 | -5 |  | G107 | NH368 | 41 |  |
| G16 | NH697 | 290 | + | G62 | NH701 | -2 |  | G108 | NH460 | 262 | + |
| G17 | NH686 | 293 | + | G63 | NH717 | 15 |  | G109 | NH294 | 252 | + |
| G18 | NH687 | 249 | + | G64 | NH695 | -10 |  | G110 | NH276 | 128 |  |
| G19 | NH649 | 260 | + | G65 | NH787 | 11 |  | G111 | NH430 | 292 | + |
| G20 | NH552 | 291 | + | G66 | NH744 | 39 |  | G112 | NH474 | 237 | + |
| G21 | NH772 | 206 | + | G67 | NH720 | -7 |  | G113 | NH264 | 233 | + |
| G22 | NH650 | 249 | + | G68 | NH755 | 12 |  | G114 | NH192 | 181 | + |
| G23 | NH709 | 262 | + | G69 | NH579 | 83 |  | G115 | NH438 | 252 | + |
| G24 | NH759 | 208 | + | G70 | NH562 | 77 |  | G116 | NH376 | 0 |  |
| G25 | NH574 | 169 | + | G71 | NH767 | 151 | + | G117 | NH207 | 244 | + |
| G26 | NH685 | 79 |  | G72 | NH774 | 98 |  | G118 | NH270 | 49 |  |
| G27 | NH652 | 266 | + | G73 | NH690 | 7 |  | G119 | NH516 | 233 | + |
| G28 | NH727 | 176 | + | G74 | NH680 | 109 |  | G120 | NH282 | 196 | + |
| G29 | NH764 | 30 |  | G75 | NH647 | 89 |  | G121 | NH185 | 146 | + |
| G30 | NH671 | 138 |  | G76 | NH648 | 181 | + | G122 | NH519 | 69 |  |
| G31 | NH661 | 52 |  | G77 | NH408 | 31 |  | G123 | NH209 | 63 |  |
| G32 | NH637 | 120 |  | G78 | NH484 | 26 |  | G124 | NH253 | 77 |  |
| G33 | NH659 | 63 |  | G79 | NH479 | 71 |  | G125 | NH210 | 127 |  |
| G34 | NH639 | 139 |  | G80 | NH494 | 83 |  | G126 | NH177 | 169 | + |
| G35 | NH656 | 31 |  | G81 | NH493 | 65 |  | G127 | NH182 | 181 | + |
| G36 | NH704 | 237 | + | G82 | NH547 | 229 | + | G128 | NH180 | 122 |  |
| G37 | NH678 | 61 |  | G83 | NH429 | 89 |  | G129 | NH91 | 83 |  |
| G38 | NH778 | 47 |  | G84 | NH544 | 45 |  | G130 | NH178 | 252 | + |
| G39 | NH653 | 128 |  | G85 | NH546 | 161 | + | G131 | NH185 | 114 |  |
| G40 | NH682 | 225 | + | G86 | NH444 | 103 |  | G132 | NH176 | 143 |  |
| G41 | NH580 | 25 |  | G87 | NH540 | 176 | + | G133 | NH213 | 222 | + |
| G42 | NH655 | 61 |  | G88 | NH412 | 205 | + | G134 | NH181 | 211 | + |
| G43 | NH737 | 42 |  | G89 | NH428 | 211 | + | G135 | NH501 | 130 |  |
| G44 | NH646 | 241 | + | G90 | NH542 | 270 | + | G136 | NH299 | 114 |  |
| G45 | NH783 | 287 | + | G91 | NH506 | 120 |  | G137 | NH60 | 259 | + |
| G46 | NH675 | 77 |  | G92 | NH424 | 1 |  | G138 | NH61 | 211 | + |
| G139 | NH48 | 289 | + | G186 | NH302 | 169 | + | G233 | NH334 | 34 |  |
| G140 | NH47 | 79 |  | G187 | NH263 | 161 | + | G234 | NH340 | 103 |  |
| G141 | NH28 | 208 | + | G188 | NH356 | 151 | + | G235 | NH399 | 15 |  |
| G142 | NH113 | 196 | + | G189 | NH265 | 41 |  | G236 | NH328 | 22 |  |
| G143 | NH100 | 135 |  | G190 | NH413 | 93 |  | G237 | NH390 | 169 | + |
| G144 | NH107 | 93 |  | G191 | NH514 | 229 | + | G238 | NH333 | 161 | + |
| G145 | NH106 | 143 |  | G192 | NH517 | 87 |  | G239 | NH332 | 71 |  |
| G146 | NH645 | 26 |  | G193 | NH266 | 169 | + | G240 | NH338 | 60 |  |
| G147 | NH670 | 52 |  | G194 | NH504 | 181 | + | G241 | NH254 | 103 |  |
| G148 | NH664 | 181 | + | G195 | NH491 | 55 |  | G242 | NH312 | 135 |  |
| G149 | NH676 | 61 |  | G196 | NH500 | 176 | + | G243 | NH311 | 161 | + |
| G150 | NH40 | 83 |  | G197 | NH290 | 98 |  | G244 | NH331 | 181 | + |
| G151 | NH462 | 229 | + | G198 | NH503 | 103 |  | G245 | NH329 | 218 | + |
| G152 | NH406 | 222 | + | G199 | NH252 | 157 | + | G246 | NH323 | 83 |  |
| G153 | NH403 | 274 | + | G200 | NH249 | 128 |  | G247 | NH320 | 146 | + |
| G154 | NH405 | 120 |  | G201 | NH212 | 181 | + | G248 | NH322 | 63 |  |
| G155 | NH404 | 181 | + | G202 | NH184 | 52 |  | G249 | NH228 | 158 | + |
| G156 | NH548 | 89 |  | G203 | NH86 | 34 |  | G250 | NH229 | 164 | + |
| G157 | NH502 | 35 |  | G204 | NH89 | 134 |  | G251 | NH323 | 69 |  |
| G158 | NH431 | 11 |  | G205 | NH211 | 168 | + | G252 | NH324 | 18 |  |
| G159 | NH434 | 34 |  | G206 | NH90 | 143 |  | G253 | NH307 | 175 | + |
| G160 | NH445 | 116 |  | G207 | NH186 | 203 | + | G254 | NH233 | 15 |  |
| G161 | NH443 | 195 | + | G208 | NH310 | 151 | + | G255 | NH319 | 4 |  |
| G162 | NH370 | 114 |  | G209 | NH351 | 0 |  | G256 | NH314 | 134 |  |
| G163 | NH454 | 69 |  | G210 | NH241 | 69 |  | G257 | NH318 | 122 |  |
| G164 | NH456 | 215 | + | G211 | NH259 | 83 |  | G258 | NH223 | 208 | + |
| G165 | NH436 | 120 |  | G212 | NH52 | 273 | + | G259 | NH224 | 103 |  |
| G166 | NH374 | 3 |  | G213 | NH50 | 229 | + | G260 | NH244 | 222 | + |
| G167 | NH541 | 154 | + | G214 | NH97 | 175 | + | G261 | NH226 | 208 | + |
| G168 | NH507 | 109 |  | G215 | NH56 | 237 | + | G262 | NH147 | 143 |  |
| G169 | NH458 | 157 | + | G216 | NH26 | 200 | + | G263 | NH18 | 120 |  |
| G170 | NH461 | 11 |  | G217 | NH111 | 228 | + | G264 | NH8 | 229 | + |
| G171 | NH418 | 119 |  | G218 | NH109 | 252 | + | G265 | NH222 | 249 | + |
| G172 | NH409 | 83 |  | G219 | NH122 | 47 |  | G266 | NH236 | 266 | + |
| G173 | NH359 | 147 | + | G220 | NH35 | 53 |  | G267 | NH220 | 211 | + |
| G174 | NH439 | 252 | + | G221 | NH563 | 98 |  | G268 | NH240 | 200 | + |
| G175 | NH415 | 38 |  | G222 | NH376 | 42 |  | G269 | NH245 | 51 |  |
| G176 | NH363 | 229 | + | G223 | NH642 | 7 |  | G270 | NH238 | 19 |  |
| G177 | NH347 | 139 |  | G224 | NH572 | 0 |  | G271 | NH239 | 98 |  |
| G178 | NH357 | 30 |  | G225 | NH36 | 5 |  | G272 | NH237 | 120 |  |
| G179 | NH426 | 12 |  | G226 | NH30 | 65 |  | G273 | NH219 | 286 | + |
| G180 | NH381 | 257 | + | G227 | NH397 | 185 | + | G274 | NH152 | 275 | + |
| G181 | NH341 | 215 | + | G228 | NH326 | 214 | + | G275 | NH216 | 296 | + |
| G182 | NH342 | 71 |  | G229 | NH327 | 177 | + | G276 | NH218 | 275 | + |
| G183 | NH293 | 15 |  | G230 | NH325 | 97 |  | G277 | NH168 | 275 | + |
| G184 | NH477 | 69 |  | G231 | NH357 | 22 |  | G278 | NH215 | 220 | + |
| G185 | NH362 | 0 |  | G232 | NH335 | 4 |  | G279 | NH167 | 130 |  |
| G280 | NH247 | 89 |  |  |  |  |  |  |  |  |  |
| G281 | NH163 | 252 | + |  |  |  |  |  |  |  |  |
| G282 | NH214 | 273 | + |  |  |  |  |  |  |  |  |
| G283 | NH157 | 278 | + |  |  |  |  |  |  |  |  |
| G284 | NH162 | 237 | + |  |  |  |  |  |  |  |  |
| G285 | NH146 | 139 |  |  |  |  |  |  |  |  |  |
| G286 | NH138 | 220 | + |  |  |  |  |  |  |  |  |
| G287 | NH85 | 299 | + |  |  |  |  |  |  |  |  |
| G288 | NH172 | 281 | + |  |  |  |  |  |  |  |  |
| G289 | NH23 | 260 | + |  |  |  |  |  |  |  |  |
| G290 | NH137 | 181 | + |  |  |  |  |  |  |  |  |
| G291 | NH110 | 181 | + |  |  |  |  |  |  |  |  |
| G292 | NH115 | 237 | + |  |  |  |  |  |  |  |  |
| G293 | NH20 | 196 | + |  |  |  |  |  |  |  |  |
| G294 | NH45 | 211 | + |  |  |  |  |  |  |  |  |
| G295 | NH175 | 299 | + |  |  |  |  |  |  |  |  |
| G296 | NH41 | 176 | + |  |  |  |  |  |  |  |  |
| G297 | NH84 | 294 | + |  |  |  |  |  |  |  |  |
| G298 | NH33 | 244 | + |  |  |  |  |  |  |  |  |
| G299 | NH25 | 286 | + |  |  |  |  |  |  |  |  |
| G300 | NH170 | 248 | + |  |  |  |  |  |  |  |  |
| G301 | NH123 | 270 | + |  |  |  |  |  |  |  |  |

Superior Mutants are lines with higher value for the trait with significant stability

| Tiller number Mean: 9.87 |
| --- |
| YS Mean: 143.17 |
| LSD (0.05): 1.19 |

**Supplementary Table 4C**. YSi statistic for plant height in 300 mutants

| Code | Mutant | YSi (Y+S) | Stable mutant with higher value  (+) | Code | Mutant | YSi (Y+S) | Stable mutant with higher value  (+) | Code | Mutant | YSi (Y+S) | Stable mutant with higher value  (+) |
| --- | --- | --- | --- | --- | --- | --- | --- | --- | --- | --- | --- |
| G1 | N22 | 175 | + | G47 | NH677 | 94 |  | G93 | NH423 | 109 |  |
| G2 | NH753 | 251 | + | G48 | NH673 | 103 |  | G94 | NH543 | 290 | + |
| G3 | NH577 | 215 | + | G49 | NH743 | 117 |  | G95 | NH410 | 144 |  |
| G4 | NH779 | 122 |  | G50 | NH681 | 79 |  | G96 | NH419 | 144 |  |
| G5 | NH699 | 245 | + | G51 | NH729 | 237 | + | G97 | NH380 | 214 | + |
| G6 | NH710 | 241 | + | G52 | NH738 | 198 | + | G98 | NH360 | 242 | + |
| G7 | NH777 | 292 | + | G53 | NH761 | 81 |  | G99 | NH427 | 263 | + |
| G8 | NH765 | 228 | + | G54 | NH556 | 208 | + | G100 | NH361 | 280 | + |
| G9 | NH756 | 240 | + | G55 | NH558 | 58 |  | G101 | NH425 | 201 | + |
| G10 | NH757 | 281 | + | G56 | NH549 | 96 |  | G102 | NH510 | 271 | + |
| G11 | NH766 | 276 | + | G57 | NH575 | 133 |  | G103 | NH346 | 248 | + |
| G12 | NH636 | 281 | + | G58 | NH651 | 39 |  | G104 | NH348 | 271 | + |
| G13 | NH662 | 211 | + | G59 | NH553 | 120 |  | G105 | NH355 | 265 | + |
| G14 | NH551 | 195 | + | G60 | NH698 | 117 |  | G106 | NH414 | 284 | + |
| G15 | NH648 | 279 | + | G61 | NH683 | 12 |  | G107 | NH368 | 300 | + |
| G16 | NH697 | 259 | + | G62 | NH701 | 174 | + | G108 | NH460 | 290 | + |
| G17 | NH686 | -5 |  | G63 | NH717 | 144 |  | G109 | NH294 | 276 | + |
| G18 | NH687 | 163 | + | G64 | NH695 | -8 |  | G110 | NH276 | 191 | + |
| G19 | NH649 | 266 | + | G65 | NH787 | 56 |  | G111 | NH430 | 292 | + |
| G20 | NH552 | 267 | + | G66 | NH744 | 24 |  | G112 | NH474 | 140 |  |
| G21 | NH772 | 230 | + | G67 | NH720 | -2 |  | G113 | NH264 | 124 |  |
| G22 | NH650 | 132 |  | G68 | NH755 | 42 |  | G114 | NH192 | 64 |  |
| G23 | NH709 | 51 |  | G69 | NH579 | 61 |  | G115 | NH438 | 132 |  |
| G24 | NH759 | 81 |  | G70 | NH562 | 57 |  | G116 | NH376 | 155 | + |
| G25 | NH574 | -4 |  | G71 | NH767 | 48 |  | G117 | NH207 | 108 |  |
| G26 | NH685 | 69 |  | G72 | NH774 | 103 |  | G118 | NH270 | 159 | + |
| G27 | NH652 | 209 | + | G73 | NH690 | 177 | + | G119 | NH516 | 248 | + |
| G28 | NH727 | 224 | + | G74 | NH680 | 1 |  | G120 | NH282 | 72 |  |
| G29 | NH764 | 187 | + | G75 | NH647 | 101 |  | G121 | NH185 | 238 | + |
| G30 | NH671 | 258 | + | G76 | NH648 | 50 |  | G122 | NH519 | -1 |  |
| G31 | NH661 | 256 | + | G77 | NH408 | 136 |  | G123 | NH209 | 88 |  |
| G32 | NH637 | 184 | + | G78 | NH484 | 28 |  | G124 | NH253 | 133 |  |
| G33 | NH659 | 194 | + | G79 | NH479 | 41 |  | G125 | NH210 | 119 |  |
| G34 | NH639 | 232 | + | G80 | NH494 | -3 |  | G126 | NH177 | 28 |  |
| G35 | NH656 | 151 | + | G81 | NH493 | 24 |  | G127 | NH182 | 213 | + |
| G36 | NH704 | 240 | + | G82 | NH547 | 28 |  | G128 | NH180 | 238 | + |
| G37 | NH678 | 200 | + | G83 | NH429 | 26 |  | G129 | NH91 | 151 | + |
| G38 | NH778 | 294 | + | G84 | NH544 | 9 |  | G130 | NH178 | 136 |  |
| G39 | NH653 | 171 | + | G85 | NH546 | 53 |  | G131 | NH185 | 86 |  |
| G40 | NH682 | 105 |  | G86 | NH444 | -9 |  | G132 | NH176 | 88 |  |
| G41 | NH580 | 49 |  | G87 | NH540 | 19 |  | G133 | NH213 | 115 |  |
| G42 | NH655 | 174 | + | G88 | NH412 | 13 |  | G134 | NH181 | 73 |  |
| G43 | NH737 | 80 |  | G89 | NH428 | 8 |  | G135 | NH501 | 62 |  |
| G44 | NH646 | -10 |  | G90 | NH542 | 13 |  | G136 | NH299 | 163 | + |
| G45 | NH783 | 66 |  | G91 | NH506 | 273 | + | G137 | NH60 | 158 | + |
| G46 | NH675 | 209 | + | G92 | NH424 | 27 |  | G138 | NH61 | 83 |  |
| G139 | NH48 | 263 | + | G186 | NH302 | 197 | + | G233 | NH334 | 258 | + |
| G140 | NH47 | 198 | + | G187 | NH263 | 94 |  | G234 | NH340 | 226 | + |
| G141 | NH28 | 158 | + | G188 | NH356 | 181 | + | G235 | NH399 | 97 |  |
| G142 | NH113 | 170 | + | G189 | NH265 | 162 | + | G236 | NH328 | 234 | + |
| G143 | NH100 | 79 |  | G190 | NH413 | 285 | + | G237 | NH390 | 253 | + |
| G144 | NH107 | 174 | + | G191 | NH514 | 214 | + | G238 | NH333 | 170 | + |
| G145 | NH106 | 69 |  | G192 | NH517 | 148 | + | G239 | NH332 | 212 | + |
| G146 | NH645 | 119 |  | G193 | NH266 | 161 | + | G240 | NH338 | 225 | + |
| G147 | NH670 | 209 | + | G194 | NH504 | 217 | + | G241 | NH254 | 120 |  |
| G148 | NH664 | 144 |  | G195 | NH491 | 120 |  | G242 | NH312 | 256 | + |
| G149 | NH676 | 66 |  | G196 | NH500 | 185 | + | G243 | NH311 | 211 | + |
| G150 | NH40 | 44 |  | G197 | NH290 | 77 |  | G244 | NH331 | 287 | + |
| G151 | NH462 | 283 | + | G198 | NH503 | 47 |  | G245 | NH329 | 290 | + |
| G152 | NH406 | 191 | + | G199 | NH252 | 158 | + | G246 | NH323 | 219 | + |
| G153 | NH403 | 295 | + | G200 | NH249 | 138 |  | G247 | NH320 | 36 |  |
| G154 | NH405 | 101 |  | G201 | NH212 | 175 | + | G248 | NH322 | 203 | + |
| G155 | NH404 | 45 |  | G202 | NH184 | 237 | + | G249 | NH228 | 193 | + |
| G156 | NH548 | 30 |  | G203 | NH86 | 284 | + | G250 | NH229 | 236 | + |
| G157 | NH502 | 99 |  | G204 | NH89 | 229 | + | G251 | NH323 | 144 |  |
| G158 | NH431 | 60 |  | G205 | NH211 | 32 |  | G252 | NH324 | 246 | + |
| G159 | NH434 | 62 |  | G206 | NH90 | 252 | + | G253 | NH307 | 291 | + |
| G160 | NH445 | 286 | + | G207 | NH186 | 99 |  | G254 | NH233 | 182 | + |
| G161 | NH443 | 101 |  | G208 | NH310 | 171 | + | G255 | NH319 | 108 |  |
| G162 | NH370 | 108 |  | G209 | NH351 | 39 |  | G256 | NH314 | 115 |  |
| G163 | NH454 | 190 | + | G210 | NH241 | 87 |  | G257 | NH318 | 109 |  |
| G164 | NH456 | 175 | + | G211 | NH259 | 117 |  | G258 | NH223 | 86 |  |
| G165 | NH436 | 214 | + | G212 | NH52 | 34 |  | G259 | NH224 | 123 |  |
| G166 | NH374 | 112 |  | G213 | NH50 | 28 |  | G260 | NH244 | 144 |  |
| G167 | NH541 | 202 | + | G214 | NH97 | 24 |  | G261 | NH226 | 213 | + |
| G168 | NH507 | 42 |  | G215 | NH56 | 174 | + | G262 | NH147 | 36 |  |
| G169 | NH458 | 148 | + | G216 | NH26 | 92 |  | G263 | NH18 | 23 |  |
| G170 | NH461 | 76 |  | G217 | NH111 | 296 | + | G264 | NH8 | 213 | + |
| G171 | NH418 | 167 | + | G218 | NH109 | 288 | + | G265 | NH222 | 73 |  |
| G172 | NH409 | 151 | + | G219 | NH122 | 234 | + | G266 | NH236 | 127 |  |
| G173 | NH359 | 227 | + | G220 | NH35 | 206 | + | G267 | NH220 | 275 | + |
| G174 | NH439 | 180 | + | G221 | NH563 | 279 | + | G268 | NH240 | 231 | + |
| G175 | NH415 | 22 |  | G222 | NH376 | 295 | + | G269 | NH245 | 32 |  |
| G176 | NH363 | 209 | + | G223 | NH642 | 139 |  | G270 | NH238 | 9 |  |
| G177 | NH347 | 169 | + | G224 | NH572 | 184 | + | G271 | NH239 | 13 |  |
| G178 | NH357 | 77 |  | G225 | NH36 | 199 | + | G272 | NH237 | 3 |  |
| G179 | NH426 | 179 | + | G226 | NH30 | 261 | + | G273 | NH219 | 226 | + |
| G180 | NH381 | 98 |  | G227 | NH397 | 134 |  | G274 | NH152 | 255 | + |
| G181 | NH341 | 136 |  | G228 | NH326 | 64 |  | G275 | NH216 | 131 |  |
| G182 | NH342 | 72 |  | G229 | NH327 | 124 |  | G276 | NH218 | 253 | + |
| G183 | NH293 | 43 |  | G230 | NH325 | 179 | + | G277 | NH168 | 248 | + |
| G184 | NH477 | 128 |  | G231 | NH357 | 194 | + | G278 | NH215 | 45 |  |
| G185 | NH362 | 70 |  | G232 | NH335 | 251 | + | G279 | NH167 | 293 | + |
| G280 | NH247 | 259 | + |  |  |  |  |  |  |  |  |
| G281 | NH163 | 72 |  |  |  |  |  |  |  |  |  |
| G282 | NH214 | 159 | + |  |  |  |  |  |  |  |  |
| G283 | NH157 | 27 |  |  |  |  |  |  |  |  |  |
| G284 | NH162 | 22 |  |  |  |  |  |  |  |  |  |
| G285 | NH146 | -6 |  |  |  |  |  |  |  |  |  |
| G286 | NH138 | 2 |  |  |  |  |  |  |  |  |  |
| G287 | NH85 | 49 |  |  |  |  |  |  |  |  |  |
| G288 | NH172 | 140 |  |  |  |  |  |  |  |  |  |
| G289 | NH23 | 73 |  |  |  |  |  |  |  |  |  |
| G290 | NH137 | 10 |  |  |  |  |  |  |  |  |  |
| G291 | NH110 | 8 |  |  |  |  |  |  |  |  |  |
| G292 | NH115 | 39 |  |  |  |  |  |  |  |  |  |
| G293 | NH20 | 198 | + |  |  |  |  |  |  |  |  |
| G294 | NH45 | 7 |  |  |  |  |  |  |  |  |  |
| G295 | NH175 | 83 |  |  |  |  |  |  |  |  |  |
| G296 | NH41 | 7 |  |  |  |  |  |  |  |  |  |
| G297 | NH84 | 270 | + |  |  |  |  |  |  |  |  |
| G298 | NH33 | 51 |  |  |  |  |  |  |  |  |  |
| G299 | NH25 | 77 |  |  |  |  |  |  |  |  |  |
| G300 | NH170 | 64 |  |  |  |  |  |  |  |  |  |
| G301 | NH123 | 268 | + |  |  |  |  |  |  |  |  |

Plant height Mean: 78.70, YS Mean: 146, LSD (0.05): 4.19

**Supplementary table 5.** YSi statistic for Grain yield in 6 mutants in low P condition

| S. no | | Yield | Yield  Rank | Adjusted rank | | Adjusted Yield  Rank (Y) | Stability variance | Stablity  rating (S) | YSi  (Y+S) | Superior  Lines (+) |
| --- | --- | --- | --- | --- | --- | --- | --- | --- | --- | --- |
| 1 | | 0.9 | 2 | -3 | | -1 | 0.073045 | 0 | -1 |  |
| 2 | | 7.091667 | 7 | 3 | | 10 | 0.025545 | 0 | 10 | + |
| 3 | | 6.725 | 6 | 3 | | 9 | 0.132091 | -8 | 1 |  |
| 4 | | 6.191667 | 5 | 3 | | 8 | 0.084455 | 0 | 8 | + |
| 5 | | 1.016667 | 3 | -3 | | 0 | 0.0865 | 0 | 0 |  |
| 6 | | 2.35 | 4 | -3 | | 1 | 0.152682 | -8 | -7 |  |
| 7 | | 0 | 1 | -3 | | -2 | 0.064318 | 0 | -2 |  |
| Yield Mean: 3.467857 | | | |  |  |  |  |  |  |  |
| YS Mean: 1.285714 | | | |  |  |  |  |  |  |  |
| LSD (0.05): 0.08663981 | | | |  |  |  |  |  |  |  |

**Supplementary table 6**. YSi statistic for Grain yield in 6 mutants in water limited condition

| S. no | Yield | Yield  Rank | | Adjusted rank | Adjusted Yield  Rank (Y) | Stability variance | Stablity  rating (S) | YSi  (Y+S) | Superior  Lines (+) |
| --- | --- | --- | --- | --- | --- | --- | --- | --- | --- |
| 1 | 8.358333 | 3 | | -3 | 0 | 3.876424 | -8 | -8 |  |
| 2 | 24.89167 | 7 | | 3 | 10 | 9.069515 | -8 | 2 | + |
| 3 | 10.19167 | 4 | | -3 | 1 | 5.070242 | -8 | -7 |  |
| 4 | 12.24167 | 5 | | 3 | 8 | -0.10176 | 0 | 8 | + |
| 5 | 19.78333 | 6 | | 3 | 9 | 4.348379 | -8 | 1 | + |
| 6 | 1.925 | 1 | | -3 | -2 | 3.132788 | -8 | -10 |  |
| 7 | 3.441667 | 2 | | -3 | -1 | 1.966606 | -8 | -9 |  |
| Yield Mean: 11.54762 | | |  |  |  |  |  |  |  |
| YS Mean: -3.285714 | | |  |  |  |  |  |  |  |
| LSD (0.05): 0.2421165 | | |  |  |  |  |  |  |  |
|  | | |  |  |  |  |  |  |  |

**Supplementary table 7.**YSi statistic for grain yield in 6 mutants in normal condition

| S. no | Yield | Yield  Rank | | Adjusted rank | Adjusted Yield  Rank (Y) | Stability variance | Stablity  rating (S) | YSi  (Y+S) | Superior  Lines (+) |
| --- | --- | --- | --- | --- | --- | --- | --- | --- | --- |
| 1 | 11.49167 | 1 | | -3 | -2 | 0.288091 | 0 | -2 |  |
| 2 | 17.68333 | 7 | | 3 | 10 | 0.968318 | -8 | 2 | + |
| 3 | 17.35 | 6 | | 3 | 9 | 1.195591 | -8 | 1 | + |
| 4 | 15.44167 | 3 | | -2 | 1 | 3.621364 | -8 | -7 |  |
| 5 | 16.66667 | 5 | | 3 | 8 | 0.685045 | -8 | 0 | + |
| 6 | 14.375 | 2 | | -3 | -1 | 0.158818 | 0 | -1 | + |
| 7 | 16.5 | 4 | | 3 | 7 | 0.712318 | -8 | -1 | + |
| Yield Mean: 15.64405 | | |  |  |  |  |  |  |  |
| YS Mean: -1.142857 | | |  |  |  |  |  |  |  |
| LSD (0.05): 0.1655504 | | |  |  |  |  |  |  |  |

**Supplementary table 8.** YSi statistic for grain yield in 6 mutants in three environments

| S. no | Yield | Yield  Rank | | Adjusted rank | Adjusted Yield  Rank (Y) | Stability variance | Stablity  rating (S) | YSi  (Y+S) | Superior  Lines (+) |
| --- | --- | --- | --- | --- | --- | --- | --- | --- | --- |
| 1 | 11.49167 | 1 | | -3 | -2 | 0.288091 | 0 | -2 |  |
| 2 | 17.68333 | 7 | | 3 | 10 | 0.968318 | -8 | 2 | + |
| 3 | 17.35 | 6 | | 3 | 9 | 1.195591 | -8 | 1 | + |
| 4 | 15.44167 | 3 | | -2 | 1 | 3.621364 | -8 | -7 |  |
| 5 | 16.66667 | 5 | | 3 | 8 | 0.685045 | -8 | 0 | + |
| 6 | 14.375 | 2 | | -3 | -1 | 0.158818 | 0 | -1 | + |
| 7 | 16.5 | 4 | | 3 | 7 | 0.712318 | -8 | -1 | + |
| Yield Mean: 15.64405 | | |  |  |  |  |  |  |  |
| YS Mean: -1.142857 | | |  |  |  |  |  |  |  |
| LSD (0.05): 0.1655504 | | |  |  |  |  |  |  |  |

**Supplementary table 9.** Marker trait association through Single marker analysis using ICIM.v.2.0

| **Trait Name** | **Chromosome** | **Marker Name** | **LOD** | **PVE(%)** | **Add** | **Pr>.001** |
| --- | --- | --- | --- | --- | --- | --- |
| TN_N | 2 | RM423 | 7.1225 | 99.1084 | -2.5892 | *** |
| YLD_P | 6 | RM72 | 4.3203 | 47.1726 | -2.8012 | *** |
| YLD_P | 11 | RM584 | 4.3203 | 47.1726 | -2.8012 | *** |
| TN_W | 2 | RM423 | 2.5061 | 81.8902 | -2.9008 | *** |

TN_N- Tiller number at Normal condition (E3), YLD_P- Grain yield at Low P (E1), TN_W – Tiller Number at water limited(E2), LOD- = log 10 (odds ratio), PVE(%)- Percentage of phenotypic variance explained, Add- Additive effect, Pr- probability
